# Novel photochemical reactions of carbocyclic diazodiketones without elimination of nitrogen – a suitable way to N-hydrazonation of C–H-bonds

**DOI:** 10.3762/bjoc.14.200

**Published:** 2018-08-28

**Authors:** Liudmila L Rodina, Xenia V Azarova, Jury J Medvedev, Dmitrij V Semenok, Valerij A Nikolaev

**Affiliations:** 1 Department of Organic Chemistry, St-Petersburg State University, 26 University pr., 198504, Saint-Petersburg, Russia; 2 Skolkovo Institute of Science and Technology, 143026, Moscow, Skolkovo Innovation Center, 3 Nobel st., Russia

**Keywords:** C–H insertion, diazo compounds, excited state, photochemistry, Wolff rearrangement

## Abstract

The sensitized photoexcitation of 2-diazocyclopentane-1,3-diones in the presence of THF leads to the insertion of the terminal N-atom of the diazo group into the α-С–Н bond of THF, producing the associated *N*-alkylhydrazones in yields of up to 63–71%. Further irradiation of hydrazones derived from furan-fused tricyclic diazocyclopentanediones culminates in the cycloelimination of furans to yield 2-*N*-(alkyl)hydrazone of cyclopentene-1,2,3-trione. By contrast, the direct photolysis of carbocyclic diazodiketones gives only Wolff rearrangement products with up to 90–97% yield.

## Introduction

Photochemical reactions of diazocarbonyl compounds are well-known transformations in the synthesis of the diversified acyclic, carbo- and heterocyclic structures [1–5]. A direct irradiation of diazo compounds by UV light usually gives rise to nitrogen elimination and generation of carbenes [6–10] or ketenes [11–18] and their ensuing transformations. On the other hand, photochemical reactions of diazocarbonyl compounds without elimination of dinitrogen are essentially limited to their isomerization into α-ketodiazirines [19–26] which is usually observed upon irradiation of diazo compounds with longer wavelength UV light.

Recently a new light-induced reaction of diazo compounds with retention of the diazo group nitrogen atoms in the structure of the reaction products was discovered by our group. The sensitized photoexcitation of heterocyclic diazoketones – diazotetrahydrofuranones resulted in the formation of *N*-alkyl-substituted hydrazones and other nitrogen-containing compounds [27–30]. This photochemical process is assumed to proceed through the triplet excited states of diazoketones via ‘insertion’ of the terminal nitrogen atom of the diazo group into the С–Н bond of the organic substrates [27,30].

Hence it was principally demonstrated that by carefully varying the irradiation conditions one can direct the photochemical reaction of diazo compounds at the alternative way, which provides the retention of ‘dinitrogen’ in the structure of molecule formed. It is reasonable that a question arises on the scope and limitations of this new light-induced reaction of diazo compounds.

**Figure 1:**
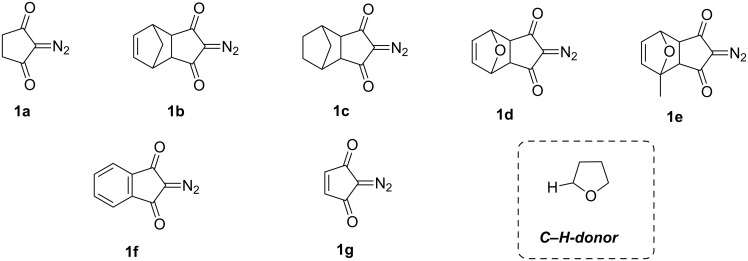
The structures of carbocyclic diazodiketones **1a–g** and C–H-donating tetrahydrofuran used in the project.

The main objective of our current research was to elucidate the possibility of using carbocyclic diazodiketones in this photochemical process. For this purpose, diazocyclopentanediones **1a–g** were tested in the study including unsubstituted diazocyclopentanedione **1a**, tricyclic diazodiketones **1b–e** with CH_2_- and O-bridges in their structure, diazoindandione **1f**, diazocyclopentenedione **1g**, and as a С–Н donor tetrahydrofuran was employed in the study (Figure 1).

To determine the most efficient conditions for this reaction with diazodiketones **1**, three sensitizers, acetophenone, benzophenone, and Michler’s ketone, with different levels of triplet state energy were tested. Furthermore, to clearly demonstrate the difference of light-induced reactions of diazo compounds **1** in the triplet and singlet excited states, the direct photolysis of diazodiketones **1a-c** without sensitizers was also examined.

## Results and Discussion

The starting diazocyclopentanediones (DDK, **1a–f**) were synthesized from the corresponding 1,3-dicarbonyl compounds [31–33] employing a diazotransfer reaction [34–39]. Diazocyclopentenedione **1g** was prepared by thermal cycloelimination from diazodiketones **1d**,**e** [38–39]. The structures of diazodiketones **1a–g** were established using a standard set of spectroscopic methods (^1^Н and ^13^С NMR, IR and UV; for details, see Supporting Information File 1, Table S1) and in the case of the tricyclic diazodiketone **1с** the structure was also confirmed by means of X-ray analysis (Supporting Information File 1, Figure S1). As it is evident from these data, the tricyclic diazodiketones **1b–e** all have *endo*-configuration of the molecular structure.

Analysis of the UV spectra of diazodiketones **1** shows that they have four absorption bands: two intense ones at 216–222 and 248–250 nm, and two bands with very week intensities at 311–316 nm (215 < ε < 290) and 363–367 nm (30 < ε < 49) (see Supporting Information File 1, Table S1). By analogy with the literature data [40], the absorption bands in the short wavelength region (216–250 nm) are attributed to π–π* electronic transitions, whereas the long wavelength bands are most likely caused by n–π* transitions. Based on the position of the long wavelength bands (363–367 nm) the energy of the singlet excited state ^1^S_1_ of diazodiketones **1** can be estimated at about 78–79 kcal/mol [41].

The characteristics of the absorption bands of the sensitizers used in this study (acetophenone, benzophenone and Michler’s ketone) demonstrate that they show appropriate absorption of the actinic light at the irradiation conditions of diazodiketones **1** (Supporting Information File 1, Table S2).

The sensitized photoreactions of diazodiketones **1a–g** were carried out in THF solutions with added sensitizers (Table 1). In the case of diazodiketone **1а** the only isolated product was hydrazone **2а** with 33–49% yields (Table 1, entries 1–3). Increasing the amount of sensitizer (up to 10:1) enhanced the yield of hydrazone **2а** by 1/2 while reducing the irradiation time by 50% (Table 1, entries 1 and 3).

**Table 1:** Sensitized light-induced reactions of diazodiketones **1а–f**.^a^

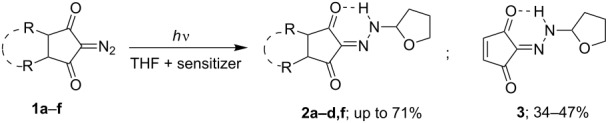					

Entry	DDK^b^	Sensitizer	Ratio sensitizer/DDK	Time, h	Yields of **2a–d**,**f** and **3** [%]

1			2:1	1.5	**2a**; 33
2	**1a**	Ph_2_CO	4:1	0.75	**2a**; 42
3			10:1	0.75	**2a**; 49

4		Ph_2_CO	1:1	1.3	**2b**; 32
5^c^		Ph_2_CO	1:1	6.5	**2b**; 34
6	**1b**	Ph_2_CO	4:1	2	**2b**; 52
7		Ph_2_CO	10:1	1.5	**2b**; 71
8		MePhCO	4:1	1.5	**2b**; 40
9		M K^d^	2:1	3.5	n.d.^e^

10	**1c**	Ph_2_CO	10:1	0.8	**2c**; 63

11	**1d**	Ph_2_CO	4:1	0.5	**2d**; 35 + **3**; 47 (total yield 82%)

12	**1e**	Ph_2_CO	4:1	1.0	**3**; 34

13	**1f**	Ph_2_CO	4:1	0.8	**2f**; 52

^a^Irradiation at λ > 210 nm. ^b^Diazodiketone. ^c^Irradiation at λ > 310 nm. ^d^Michler’s ketone. ^e^Inseparable complex reaction mixture was obtained with Michler’s ketone.

**Figure 2:**
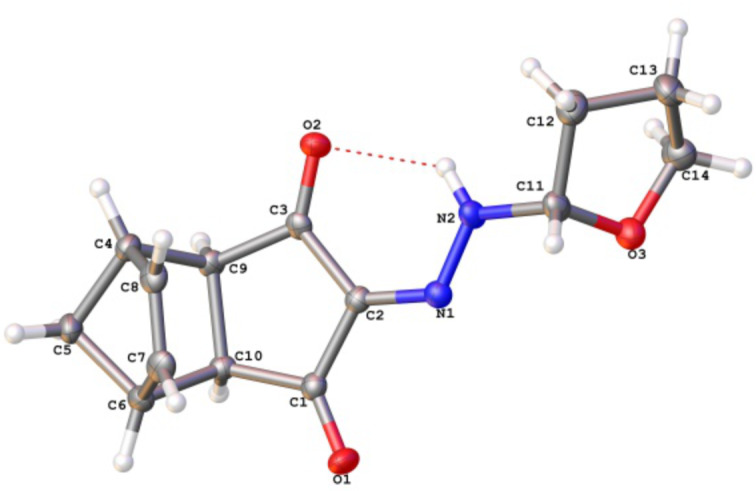
Molecular structure of hydrazone **2b** as determined by X-ray analysis data (Olex2 plot with 50% probability level of ellipsoids).

The irradiation of tricyclic diazodiketone **1b** in the presence of 1 equiv of benzophenone in THF solution led to the formation of hydrazone **2b** in a yield of 32% (Table 1, entry 4), whose structure was unambiguously established by X-ray analysis (Figure 2).

To increase the yields of hydrazones **2**, an effort was undertaken to optimize the photochemical reaction conditions by varying the wavelength of irradiation, sensitizer, as well as the ratio of sensitizer/diazodiketone **1b** (Table 1, entries 4–9).

As can be seen from the data in Table 1 the most effective sensitizer for the light-induced reaction studied in the series of acetophenone, benzophenone, and Michler’s ketone was found to be benzophenone. The application of acetophenone reduced the yield of hydrazone **2b** by about 1/4 as compared to benzophenone (from 52 to 40%; Table 1, entries 6 and 8). With Michler’s ketone, a complex product mixture was obtained, from which it was impossible to isolate pure compounds.

When varying the ratio of sensitizer/diazodiketone **1b**, the best result was obtained with a 10-fold excess of benzophenone (Table 1, entry 7; 71% yield). Application of a 4-fold excess of this sensitizer diminished the yield of hydrazone **2b** to 52% (Table 1, entry 6). In going from short wavelength (λ > 210 nm) to longer wavelength (λ > 310 nm) irradiation, the yield of hydrazone **2b** remained practically unchanged, however the reaction time considerably increased from 1.3 to 6.5 h; Table 1, entries 4 and 5).

The reactions of tricyclic diazodiketone **1c** and diazoindandione **1f** in the presence of benzophenone as sensitizer proceeded in a similar way to diazo compound **1b** under the optimal conditions affording hydrazones **2c**,**f** in yields of 63 and 52%, respectively (Table 1, entries 10 and 13).

**Scheme 1:**
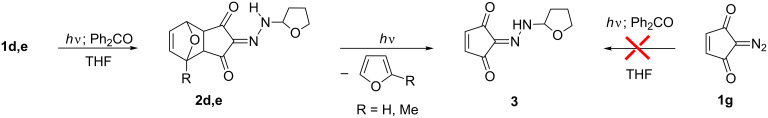
Photochemical cycloelimination of furans from hydrazones **2d**,**e**.

An unexpected side reaction was observed during irradiation of furan-fused tricyclic diazodiketones **1d,e** (Scheme 1). In the case of diazodiketone **1d**, hydrazone **3** (47%) was isolated from reaction mixture in addition to the expected hydrazone **2d** (35%; total yield 82%, Table 1, entry 11), whereas the same reaction with diazodiketone **1e** afforded hydrazone **3** exclusively (Table 1, entry 12).

Additional experiments showed that hydrazone **3** was not formed on sensitized photoexcitation of diazocyclopentenedione **1g** in the presence of the C–H-donor THF (Scheme 1). Thus one can conclude that product **3** is generated by a light-induced cycloelimination of furan from the initially generated tricyclic hydrazones **2d**,**e**.

Hence it was established that the sensitized photoexcitation of diazodiketones **1a–d,f** in THF solution proceeds through the insertion of the terminal N-atom of the diazo group in the α-С–Н-bond of THF with the formation of the appropriate hydrazones **2a–d**,**f** and the most effective sensitizer for this reaction was found to be benzophenone.

The direct photolysis of diazodiketones **1a–c** was carried out by UV light (λ > 210 nm) in THF solution containing a small amount of H_2_O or MeOH to trap the proposed intermediate α-oxoketene. These light-induced processes with diazodiketones **1a–c** resulted in formation of 2-oxocyclobutanecarboxylic acid **4a** or methyl esters **5a–c** in high isolated yields of up to 90% (Table 2). At the same time no insertion products of the possible intermediate dioxocarbenes into O–H-bonds of the nucleophilic reagents (H_2_O, MeOH) or C–H-bonds at С^5^ or С^6^ atoms in **1** [26,42–45] were detected in the reaction mixtures. Thus, it was experimentally shown that the direct photolysis of diazodiketones **1а–c** employing UV light with λ > 210 nm produces only Wolff rearrangement products in high yields.

**Table 2:** Direct photolysis of diazodiketones **1a–с** by UV light with λ > 210 nm.

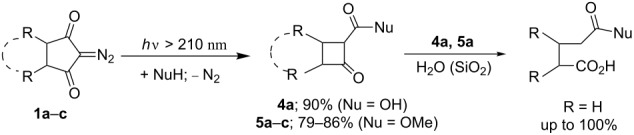				

Entry	DDK **1a–c**	Solvent/NuH (ratio)	Time, min	Yields of **4**,**5** [%]^a^

1	**1a**	THF/Н_2_О (45:1)	50	**4a**; 90
2	**1a**	THF/МеОН (20:1)	55	**5a**; 86

3	**1b**	THF/МеОН (200:1)	55	**5b**; 79 (97)

4	**1c**	THF/МеОН (50:1)	45	**5с**; 82

^a^Yield determined by ^1^H NMR spectroscopy is given in parenthesis.

The structure of 2-oxocyclobutanecarboxylic acid **4a** and the methyl esters **5a–c** was established by spectroscopic data (^1^Н, ^13^С NMR, and HRMS), and in case of **4a** and **5a,** also confirmed by comparison with the known literature data [46–48].

It was found that 2-oxocyclobutanecarboxylic acid **4a** and ester **5a** were easily hydrolyzed in the presence of H_2_O or during chromatography on silica gel to produce glutaric acid and the corresponding methyl ester (Table 2). By contrast, esters **5b**,**c** proved to be much more stable to hydrolysis on SiO_2_ and in the presence of nucleophilic reagents. For example, when heating in boiling MeOH for 2 h no hydrolysis of ketoester **5с** occurs according to ^1^H NMR spectra.

**Scheme 2:**
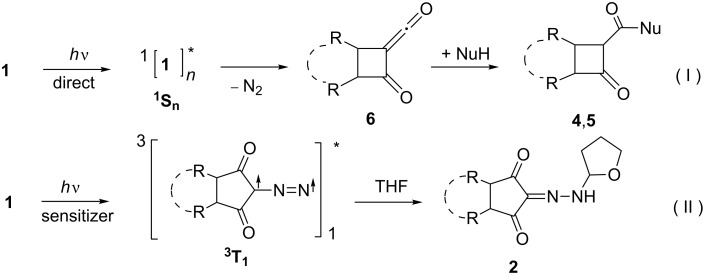
Different pathways of diazodiketones **1** light-induced reactions in the singlet (reaction I) and triplet (reaction II) excited states.

The assumed ways for light-induced reactions of diazodiketones **1** can be represented as follows. The direct irradiation (λ > 210 nm) of diazodiketones **1** gives rise to accumulation of one of their upper singlet excited states ^1^(**1**)* [13,15,18,20,49–51] which turn into usual Wolff rearrangement products, i.e., highly reactive oxoketenes **6** (Scheme 2, reaction I). The intermediate ketenes **6** react with H_2_O or MeOH present in the reaction mixture to give 2-oxocarboxylic acid **4a** or esters **5a–c**.

On the other hand, the sensitized photoexcitation of diazodiketones **1** gives rise to generation of triplet excited states of diazo compounds ^3^(**1**)_1_ which interact with the H-donor (tetrahydrofuran) producing N-alkyl-substituted hydrazones **2** (Scheme 2, reaction II). Based on the comparison study of the sensitizers’ efficiency in this reaction (Table 2) one can conclude [41,52], that the energy of the triplet excited states ^3^T_1_ of diazodiketones **1** are about 68 kcal/mol. This provides a possible explanation why the use of Michler’s ketone was found to be ineffective as a photosensitizer in the process. The energy of the triplet excited state ^3^T_1_ of this sensitizer (64 kcal/mol) is somewhat lower than the corresponding energy of the diazodiketones **1** making it inadequate to initiate the C–H insertion reaction. On the other hand, the triplet excited state energy of benzophenone (69 kcal/mol) renders it a suitable sensitizer for this reaction.

In the case of tricyclic diazodiketones **1d**,**f** (with annulated furan and 2-methylfuran-motifs in their structures), a tandem photochemical process of C–H-insertion followed by cycloelimination of furan from the initially generated hydrazones **2d**,**f** apparently occurs, giving rise to the formation of hydrazone **3** as the final reaction product (Scheme 1).

On direct irradiation of tricyclic diazodiketones **1** possessing *endo*-configuration (Figure 2), one would expect in addition to the Wolff rearrangement also the occurrence of an intramolecular cyclopropanation (with **1b**,**d**,**e**) [53–55] or an insertion of the assumed intermediate dioxocarbenes in C–H-bonds at С^5^ or С^6^-atoms (with **1c**) [26,42–45]. However, the performed experiments did not confirm the formation of corresponding reaction products in the photochemical reactions studied. This can be considered as an argument in favor of the concerted Wolff rearrangement [11–18] in the case of these diazodiketones.

## Conclusion

The sensitized photoexcitation of carbocyclic diazodiketones proceeds without elimination of dinitrogen. Instead, the reaction leads to the insertion of the terminal N-atom of the diazo group into the C–H bond of THF giving rise to the appropriate N-alkyl-substituted hydrazones in yields of up to 63–71%. The most powerful sensitizer for this light-induced process in the series of acetophenone, benzophenone and Michler’s ketone was found to be benzophenone. Hence, the diazodicarbonyl compounds can be successfully used for a photochemical N-functionalization of C–H-bonds of organic compounds. In contrast to this, the direct photolysis of carbocyclic diazoketones produces exclusively Wolff rearrangement products in yields of 90–97%.

## Experimental

### General methods

All reactions were carried out under argon atmosphere in solvents that were purified and dried before use by common methods. Monitoring of the reaction course was accomplished by thin layer chromatography (TLC) on silica gel SIL G/UV254 plates (Marcherey, Nagel & Co.). Chromatography was performed using Merck silica gel 60, 230–400 mesh. ^1^H and ^13^C NMR spectra were recorded using a Bruker-400 Avance NMR spectrometer. All HRMS spectra were recorded on a «MaXis» (Bruker Daltonik GmbH), the HPLC system (UHPLC) with the combined high-resolution quadrupole-time-of-flight mass spectrometer with electrospray ionization (ESI-QTOF). Chemical shifts are reported in ppm, and coupling constants are given in hertz (Hz). All signals in NMR spectra were normalized relative to signals of CНCl_3_ (δ = 7.26 ppm in ^1^H NMR) and CDCl_3_ (δ = 77.0 in ^13^C NMR spectra).

For single crystal X-ray diffraction experiments of **1c** (CCDC 1584937) and **2b** (CCDC 1584938) an Agilent Technologies «Xcalibur» diffractometer with monochromated MoKα radiation was used. All samples were measured at 100 K. The unit cell parameters were refined by least square techniques in CrysAlisPro (Agilent Technologies, 2012) program complex. Empirical absorption correction was applied using spherical harmonics, implemented in SCALE3 ABSPACK scaling algorithm. The structures were solved by the Superflip [56–58] and ShelXS [59] structure solution programs using Charge Flipping and Direct Methods, respectively, then refined by means of the ShelXL [60] program, incorporated in the Olex2 program package [61].

### Sensitized reactions of diazodiketones **1a–g**

**General procedure**. The solution of diazodiketone **1a–g** (1 equiv) and benzophenone (up to 10 equiv) in THF (10–30 ml) was irradiated in a quartz reactor (λ > 210 nm) under an argon atmosphere for up to 6.5 h (control of the reacted diazodiketone **1** by TLC). Thereupon the reaction mixture was dried over magnesium sulfate, the solvent completely distilled off in vacuum and the residue separated by column chromatography (SiO_2_, eluent: petroleum ether → petroleum ether/acetone 8:1) to give the hydrazones **2** and **3**.

**A. Sensitized photoexcitation of 2-diazocyclopentane-1,3-dione (1a)*.*** The reaction was performed according to the general procedure with 496 mg (4 mmol, 1 equiv) of diazodiketone **1a** and 2.912 g (16 mmol, 4 equiv) of benzophenone in 30 ml of THF with an irradiation time of 45 min. After the workup procedure hydrazone **2a** and unreacted diazoketone **1a** were isolated.

**2-(2-(Tetrahydrofuran-2-yl)hydrazono)cyclopentane-1,3-dione (2a).** Yield 168 mg (42%, calculated on the reacted diazodiketone **1a**), bright-yellow oil; ^1^H NMR (400 MHz, CDCl_3_, δ) 12.93 (s, 1H), 5.61–5.53 (m, 1H), 4.08–3.99 (m, 1H), 3.95–3.88 (m, 1H), 2.69–2.60 (m, 4H), 2.33–2.19 (m, 2H), 2.08–1.96 (m, 2H) ppm; ^13^C NMR (101 MHz, CDCl_3_, δ) 200.3, 198.7, 130.9, 92.5, 69.2, 33.3, 31.8, 31.2, 24.4 ppm; HRMS–ESI (*m*/*z*): [M + Na]^+^ calcd for C_9_H_12_N_2_O_3_, 219.0746; found, 219.0746.

**B. Sensitized photoexcitation of 2-diazo-3a,4,7,7a-tetrahydro-1*H*-4,7-methanoindene-1,3(2*H*)-dione (1b)*.*** The reaction was performed according to the general procedure with 380 mg (2 mmol, 1 equiv) of diazodiketone **1b** and 3.64 g (20 mmol, 10 equiv) of benzophenone in 20 ml of THF with an irradiation time of 90 min. After the standard workup procedure hydrazone **2b** was isolated.

**2-(2-(Tetrahydrofuran-2-yl)hydrazono)-3a,4,7,7a-tetrahydro-1*H*-4,7-methanoindene-1,3(2*H*)-dione (2b).** Yield 374 mg (71%); bright-yellow solid; mp 127–129 °С; ^1^H NMR (400 MHz, CDCl_3_, δ) 12.78 (s, 1H), 6.11–5.98 (m, 1H), 6.00–5.87 (m, 1H), 5.54–5.41 (m, 1H), 4.05–3.94 (m, 1H), 3.92–3.82 (m, 1H), 3.38 (d, *J* = 20.3 Hz, 2H), 3.20–3.07 (m, 2H), 2.32–2.10 (m, 2H), 2.03–1.92 (m, 2H), 1.63 (dt, *J* = 8.6, 1.5 Hz, 1H), 1.48 (d, *J* = 8.6 Hz, 1H) ppm; ^13^C NMR (101 MHz, CDCl_3_, δ) 201.3, 199.51, 199.49, 134.9, 134.8, 133.7, 133.63, 133.55, 133.53, 92.44, 92.39, 69.0, 68.9, 52.39, 52.37, 49.8, 48.2, 48.14, 46.14, 46.11, 46.04, 46.00, 31.2, 31.1, 24.4, 24.3 ppm; ^1^H NMR (400 MHz, acetone-*d*_6_, δ) 6.36–5.71 (m, 2H), 5.48 (dd, *J* = 6.6, 3.6 Hz, 1H), 4.14–3.75 (m, 2H), 3.46–3.01 (m, 5H), 2.46–2.12 (m, 2H), 2.10–1.73 (m, 2H), 1.62–1.52 (m, 2H) ppm; ^13^C NMR (101 MHz, acetone-*d*_6_, δ) 199.8, 134.1, 133.7, 92.1, 68.5, 52.0, 48.8, 45.9, 30.3, 24.4 ppm; HRMS–ESI (*m*/*z*): [M + Na]^+^ calcd for C_14_H_16_N_2_O_3_, 283.1059; found, 283.1065.

**C. Sensitized photoexcitation of 2-diazohexahydro-1*H*-4,7-methanoindene-1,3(2*H*)-dione (1c)*.*** The reaction was performed according to the general procedure with 376 mg (2 mmol, 1 equiv) of diazodiketone **1c** and 3.64 g (20 mmol, 10 equiv) of benzophenone in 20 ml of THF with an irradiation time of 1 h. After the standard workup procedure hydrazone **2c** was isolated.

**2-(2-(Tetrahydrofuran-2-yl)hydrazono)hexahydro-1*H*-4,7-methanoindene-1,3(2*H*)-dione (2c).** Yield 328 mg (63%); bright-yellow oil; ^1^H NMR (400 MHz, CDCl_3_, δ) 13.10 (broad, 1H, NH), 5.66–5.51 (m, 1H), 4.11–4.01 (m, 1H), 3.98–3.89 (m, 1H), 3.02–2.90 (m, 2H), 2.75–2.86 (m, 2H), 2.37–2.19 (m, 2H), 2.11–1.99 (m, 2H), 1.65–1.54 (m, 2H), 1.53–1.44 (m, 2H), 1.39–1.31 (m, 1H), 1.27–1.15 (m, 1H) ppm; ^13^C NMR (101 MHz, CDCl_3_, δ) 202.96, 202.95, 202.0, 201.0, 133.2, 133.0, 92.7, 92.6, 69.2, 69.1, 52.68, 52.65, 50.99, 50.98, 42.49, 42.47, 40.62, 40.59, 40.55, 40.52, 31.34, 31.26, 24.7, 24.59, 24.56, 24.5, 24.4 ppm; HRMS–ESI (*m*/*z*): [M + Na]^+^ calcd for C_14_H_18_N_2_O_3_, 285.1215; found, 285.1210.

**D. Sensitized photoexcitation of 2-diazo-3a,4,7,7a-tetrahydro-1*H*-4,7-epoxyindene-1,3(2*H*)-dione (1d).** The reaction was performed according to the general procedure with 380 mg (2 mmol, 1 equiv) of diazodiketone **1d** and 1.46 g (8 mmol, 4 equiv) of benzophenone in 20 ml of THF with an irradiation time of 0.5 h. After the standard workup procedure hydrazones **2d**, **3** and unreacted diazoketone **1d** were isolated.

**2-(2-(Tetrahydrofuran-2-yl)hydrazono)-3a,4,7,7a-tetrahydro-1*H*-4,7-epoxyindene-1,3(2*H*)-dione (2d).** Yield 134 mg (35% based on reacted diazodiketone **1d**); yellow oil; ^1^H NMR (400 MHz, CDCl_3_, δ) 12.83 (broad, 1H), 6.67–6.31 (m, 2H), 5.60–5.49 (m, 1H), 5.29 (s, 1H), 5.23 (s, 1H), 4.10–3.95 (m, 1H), 3.98–3.85 (m, 1H), 2.76–2.60 (m, 2H), 2.34–2.20 (m, 2H), 2.11–1.89 (m, 2H) ppm; ^13^C NMR (101 MHz, CDCl_3_, δ) 198.73, 198.70, 197.37, 197.36, 137.4, 136.8 133.50, 133.46, 92.7, 92.4, 82.5, 81.8, 69.25, 69.22, 51.01, 50.99, 49.51, 49.48, 31.3, 31.2, 24.3 ppm; HRMS–ESI (*m*/*z*): [M + H]^+^ calcd for C_13_H_14_N_2_O_4_, 263.1032; found, 263.1029.

**2-(2-(Tetrahydrofuran-2-yl)hydrazono)cyclopent-4-ene-1,3-dione (3).** Yield 135 mg (47%, on the reacted diazodiketone **1d**); yellow oil; ^1^H NMR (400 MHz, CDCl_3_, δ) 11.07 (s, 1H), 7.09 (d, *J* = 6.7 Hz, 1H), 7.05 (d, *J* = 6.7 Hz, 1H), 5.62–5.45 (m, 1H), 4.13–3.79 (m, 1H), 2.40–1.75 (m, 1H) ppm; ^13^C NMR (101 MHz, CDCl_3_, δ) 191.9, 189.9, 145.8, 143.5, 137.0, 91.5, 68.5, 30.7, 24.7 ppm; HRMS–ESI (*m*/*z*): [M + H]^+^ calcd for C_9_H_10_N_2_O_3_, 195.0770; found, 195.0774.

**E. Sensitized photoexcitation of 2-diazo-4-methyl-3a,4,7,7a-tetrahydro-1*H*-4,7-epoxyindene-1,3(2*H*)-dione (2e).** The reaction was performed according to the general procedure with 204 mg (2 mmol, 1 equiv) of diazoketone **1e** and 728 mg (8 mmol, 4 equiv) of benzophenone in 10 ml of THF with an irradiation time of 1 h. After the workup procedure hydrazone **3** [66 mg (34%)] was isolated exclusively.

**F. Sensitized photoexcitation of 2-diazo-1*H*-indene-1,3(2*H*)-dione (2f).** The reaction was performed according to the general procedure with 344 mg (2 mmol, 1 equiv) of diazodiketone **1f** and 1.46 g (8 mmol, 4 equiv) of benzophenone in 20 ml of THF with and irradiation time of 0.8 h. After standard workup procedure hydrazone **2f** and unreacted diazodiketone **1f** were isolated.

**2-(2-(Tetrahydrofuran-2-yl)hydrazono)-1*H*-indene-1,3(2*H*)-dione (2f).** Yield 70 mg (52%, calculated on the reacted diazodiketone **1f**); yellow oil; ^1^H NMR (400 MHz, CDCl_3_, δ) 11.79 (broad, 1H), 7.96–7.85 (m, 1H), 7.84–7.77 (m, 1H), 7.77–7.65 (m, 2H), 5.71–5.45 (m, 1H), 4.14–3.95 (m, 1H), 3.96–3.67 (m, 1H), 2.44–2.12 (m, 2H), 2.15–1.70 (m, 2H) ppm; ^13^C NMR (101 MHz, CDCl_3_, δ) 188.3, 186.4, 140.2, 138.6, 135.1, 134.8, 130.1, 123.1, 122.7, 91.9, 68.6, 31.0, 24.7 ppm; HRMS–ESI (*m*/*z*): [M + H]^+^ calcd for C_13_H_12_N_2_O_3_, 245.0926; found, 245.0926.

### Direct photolysis of diazodiketones **1a–c**

**Photolysis of diazodiketone 1a (with H_2_O).** A solution of sublimated diazodiketone **1а** (1.24 g, 0.01 mol) in 90 ml of pure anhydrous THF and distilled H_2_O (1.8 ml, 0.1 mol) was irradiated in a quartz reactor (λ > 210 nm) for 50 min until complete disappearance of diazodiketone **1a** (control by TLC). Then the reaction mixture was dried with magnesium sulfate and the solvent distilled off in vacuum. The obtained residue was dissolved in 25 ml of anhydrous Et_2_O, cooled to −75 to −78 °C. The precipitated resinous matter was separated by decantation and the solvent was completely removed in vacuum. The resulting oily residue was taken up in 2–3 ml of anhydrous Et_2_O and separated by column chromatography (5 g of anhydrous neutral SiO_2_; eluent: anhydrous diethyl ether) to give acid **4a** as a crystalline solid after usual workup and careful removal of solvent in vacuum from the proper fractions (0.5–0.1 mm Hg).

**Cyclobutane-2-one carboxylic acid (4а)** [46–47]***.*** Yield 1.03 g (90%); mp 43–44.5 °C (distilled); bp 93.5–94 °C (0.2–0.1 mm Hg). It is noteworthy that acid **4а** is a very hygroscopic compound which is easily hydrolyzed to produce glutaric acid.

**Photolysis of diazodiketone 1a (with MeOH)**. A solution of sublimated diazodiketone **1а** (2.48 g, 0.02 mol) in 80 ml of anhydrous THF and pure CH_3_OH (4 ml, 0.10 mol) was irradiated in a quartz reactor (λ > 210 nm) for 55 min and worked up similarly as described in the previous experiment. The resulting reaction mixture after removal of THF and methanol in vacuum was distilled to give methyl ester **5а**.

**Methyl cyclobutane-2-one carboxylate (5а)** [48]. Yield 2.20 g (86%); bp 43.5–45 °С (1 mm Hg); *n*_D_^20^ 1.5111. During column chromatography with SiO_2_ of I or II activity, methyl ester **5а** considerably hydrolyzes to give Me ester of glutaric acid.

**Photolysis of diazodiketone 1b (with MeOH)**. A solution of diazodiketone **1b** (141 mg, 0.75 mmol) in 10 ml of THF/CH_3_OH 200:1 was irradiated for 55 min in a quartz reactor (λ > 210 nm). Then the reaction mixture was dried with magnesium sulfate and the solvent and excess of CH_3_OH were completely removed in vacuum. The residue containing ester **5b** (140 mg; 97% by ^1^H NMR) was separated by preparative TLC to furnish Me ester **5b**.

**Methyl 4-oxotricyclo[4.2.1.02,5]non-7-ene-3-carboxylate (5b)**. Yield 114 mg (79%; 97% by ^1^H NMR); colorless oil; ^1^Н NMR (400 MHz, CDCl_3_, δ) 6.23–6.19 (m, 2H), 4.02–3.80 (m, 1H), 3.71 (s, 3H), 3.32–2.99 (m, 4H), 1.82–1.75 (m, 1H), 1.53–1.47 (m, 1H) ppm; ^13^C NMR (101 MHz, CDCl_3_, δ) 201.4, 167.8, 136.2, 133.4, 67.1, 63.9, 54.3, 52.4, 46.6, 43.9, 30.8 ppm; HRMS–ESI (*m*/*z*): [M + Na]^+^ calcd for C_11_H_12_O_3_, 215.0684; found, 215.0681.

**Photolysis of diazodiketone 1c (with MeOH)**. A solution of diazodiketone **1c** (190 mg, 1 mmol) in 10 ml of THF/MeOH 50:1 was irradiated in a quartz reactor (λ > 210 nm) for 45 min. Then the reaction mixture was dried with magnesium sulfate and the solvent and excess of CH_3_OH were completely distilled off in vacuum. The residue, containing ester **5c** (189 mg; 98% by ^1^H NMR) was separated by preparative TLC to give pure methyl ester **5c**.

**Methyl 4-oxotricyclo[4.2.1.0^2,5^]nonane-3-carboxylate (5c)**. Yield 158 mg (82%); colorless oil; ^1^Н NMR (400 MHz, CDCl_3_, δ) 3.92–3.87 (m, 1H), 3.75–3.66 (m, 4H), 3.06–2.99 (m, 1H), 2.63–2.57 (m, 1H), 2.59–2.50 (m, 1H), 1.65–1.53 (m, 5H), 1.41–1.33 (m, 1H) ppm; ^13^C NMR (101 MHz, CDCl_3_, δ) 203.7, 168.2, 69.6, 61.6, 52.5, 43.7, 40.8, 38.9, 34.9, 25.7, 24.6 ppm; HRMS–ESI (*m*/*z*): [M + Na]^+^ calcd for C_11_H_14_O_3_, 217.0841; found, 217.0835.

## Supporting Information

File 1 NMR spectra of all new compounds and data of X-ray analysis for compounds **1c** (CCDC 1584937) and **2b** (CCDC 1584938).
