# Supplementary material for: Novel photochemical reactions of carbocyclic diazodiketones without elimination of nitrogen – a suitable way to N-hydrazonation of C–H-bonds
Source: Beilstein J Org Chem. 2018 Aug 28;14:2250–8. doi: 10.3762/bjoc.14.200 (PMC6122312; doi:10.3762/bjoc.14.200)

Supporting Information

for

Novel photochemical reactions of carbocyclic diazodiketones without elimination of nitrogen – a suitable way to N-hydrazonation of C–H-bonds

Liudmila L. Rodina[1], Xenia V. Azarova[1], Jury J. Medvedev[1], Dmitrij V. Semenok[2] and Valerij A. Nikolaev[1]*

Address: [1]Department of Organic Chemistry, St-Petersburg State University, 26 University pr., 198504, Saint-Petersburg, Russia and [2]Skolkovo Institute of Science and Technology, 143026, Moscow, Skolkovo Innovation Center, 3 Nobel st., Russia

Email: Valerij A. Nikolaev* - valerij.nikolaev@gmail.com

*Corresponding author

NMR spectra of all new compounds and data of X-ray analysis for compounds **1c** (CCDC 1584937) and **2b** (CCDC 1584938)

Table of contents:

Molecular structure of diazodiketone **1c** S2
Characteristics of UV absorption spectra of diazodiketones **1a–c** and sensitizers S3
^{1}H and ^{13}C NMR spectra of new compounds S4
X-Ray data for compounds **1c** and **2b** S13

Molecular structure of diazoketone 1c

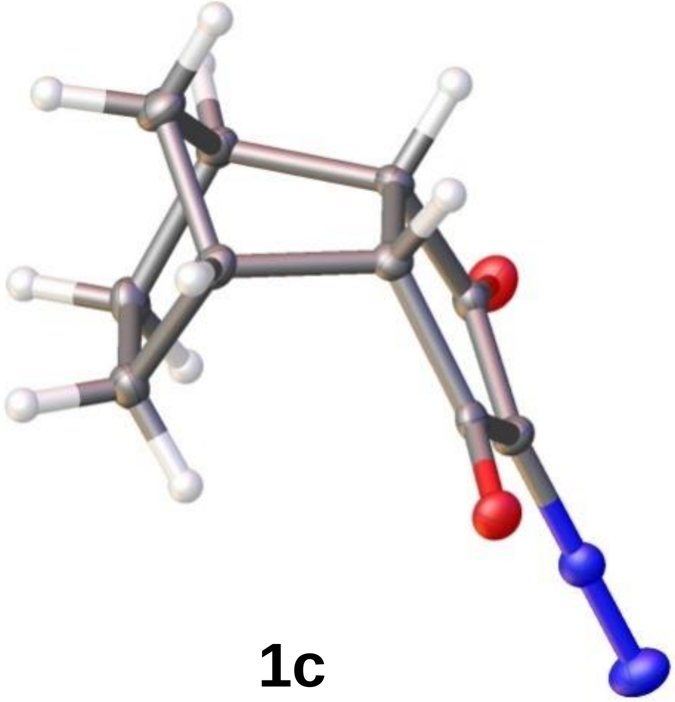

1c

Figure S1. Molecular structure of diazodiketone **1c** according to the data of X-ray analysis (Olex2 plot with 50% probability level of ellipsoids).

Characteristics of UV absorption spectra of diazodiketones 1a–c and sensitizers used

Table S1. The characteristics of the absorption bands in the UV spectra of diazodiketones **1a–c**

Diazodiketone **1a–c**	λ_{max}, nm	ε	lg (ε)
**1a**	216	14800	4,2
	248	13900	4,1
	311	260	2,4
	363	49	1,7
**1b**	222	14900	4,2
	249	19400	4,3
	316	215	2,3
	366	30	1,5
**1c**	221	16000	4,2
	250	16700	4,2
	315	290	2,5
	367	43	1,6

Table S2. Characteristics of the absorption spectra of sensitizers used

Sensitizer	λ_{max}, nm	ε	lg (ε)	E_t, (kcal/mol)
Acetophenone	240	13900	4,1	74
Benzophenone	251	27700	4,4	69
Michler's ketone	242	17400	4,2	64
	346	43000	4,6	

^{1}H and ^{13}C NMR of compound **2a**

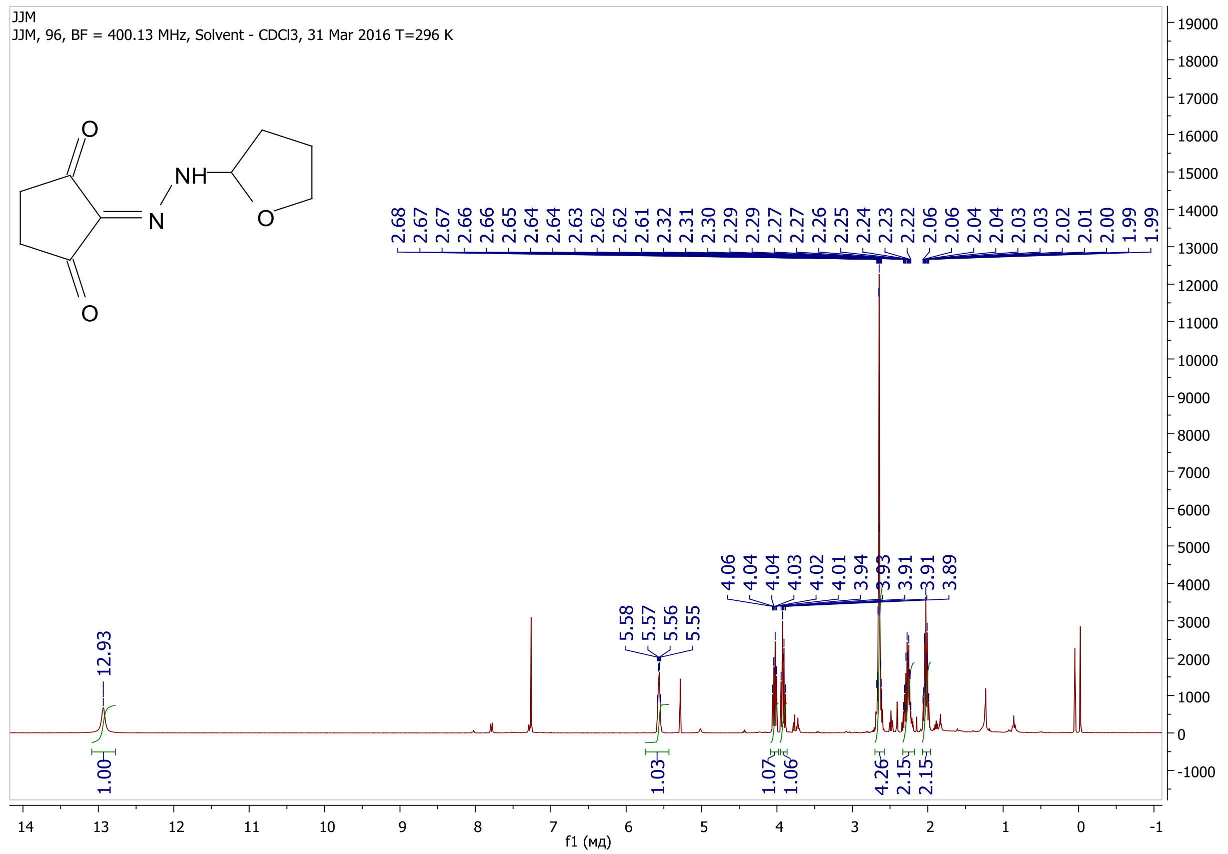

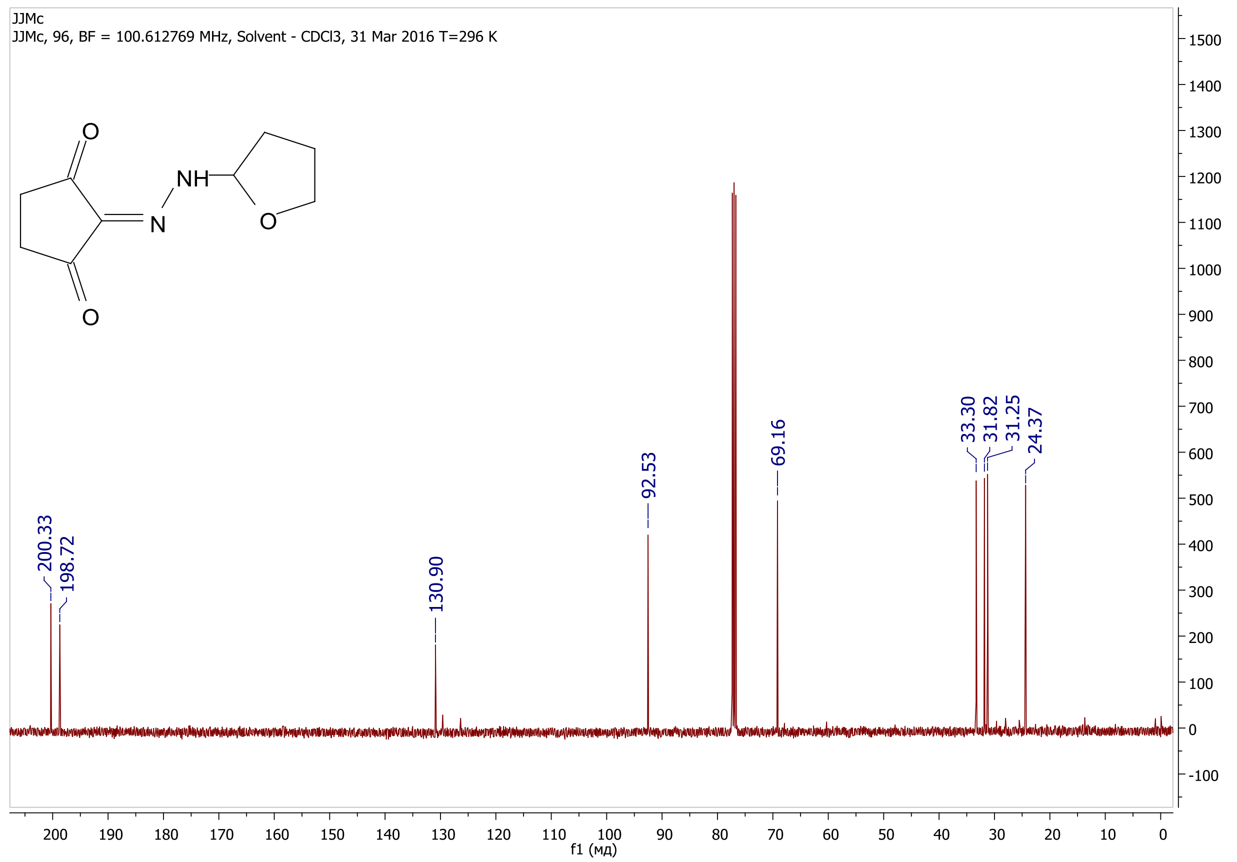

^{1}H and ^{13}C NMR of compound **2b**

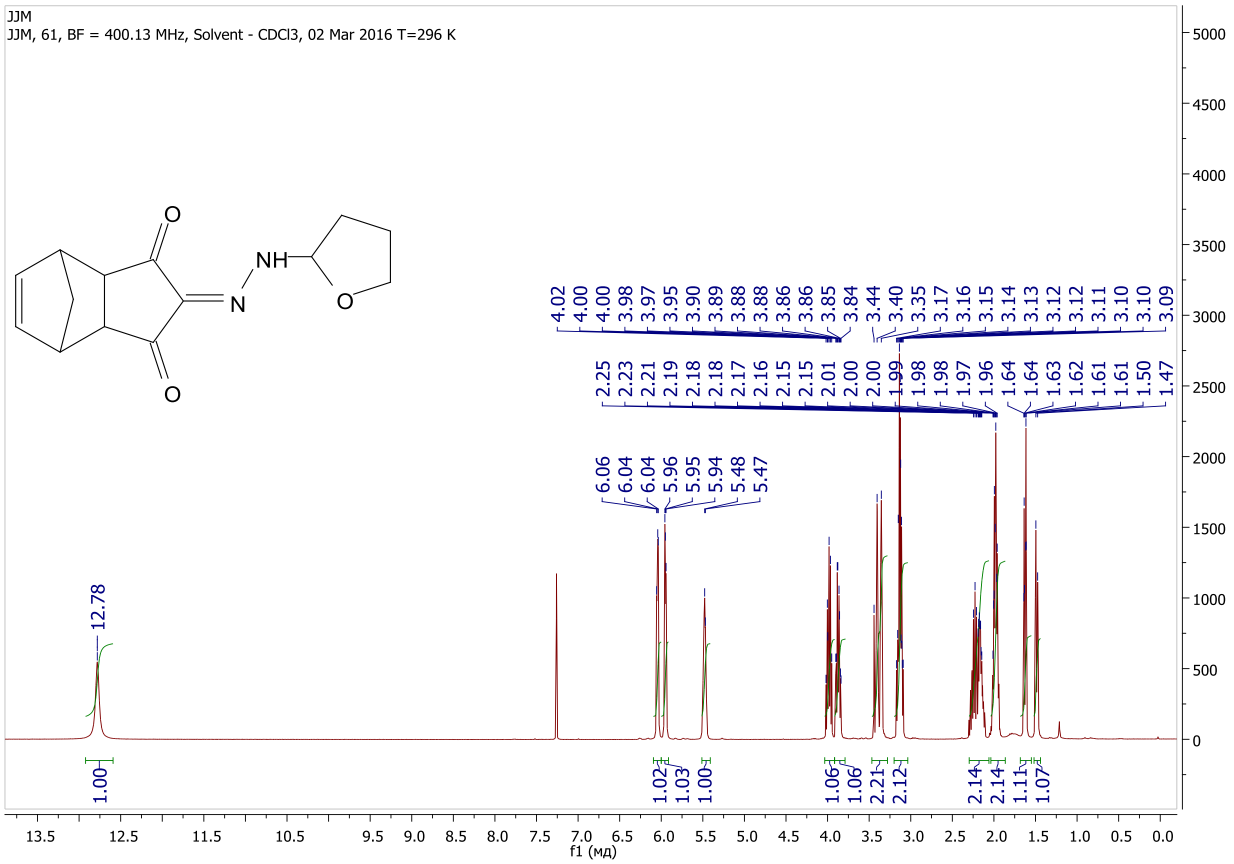

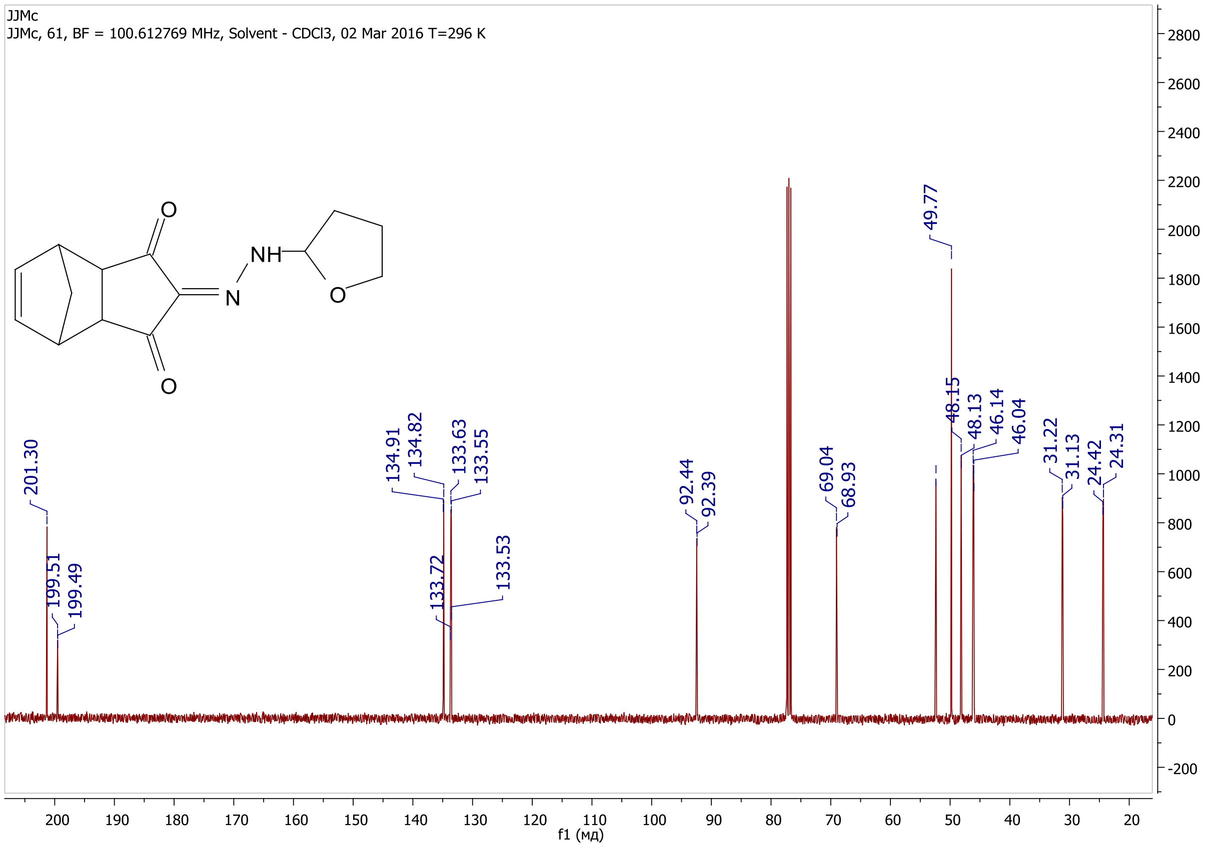

^{1}H and ^{13}C NMR of compound **2c** (in acetone-d_6)

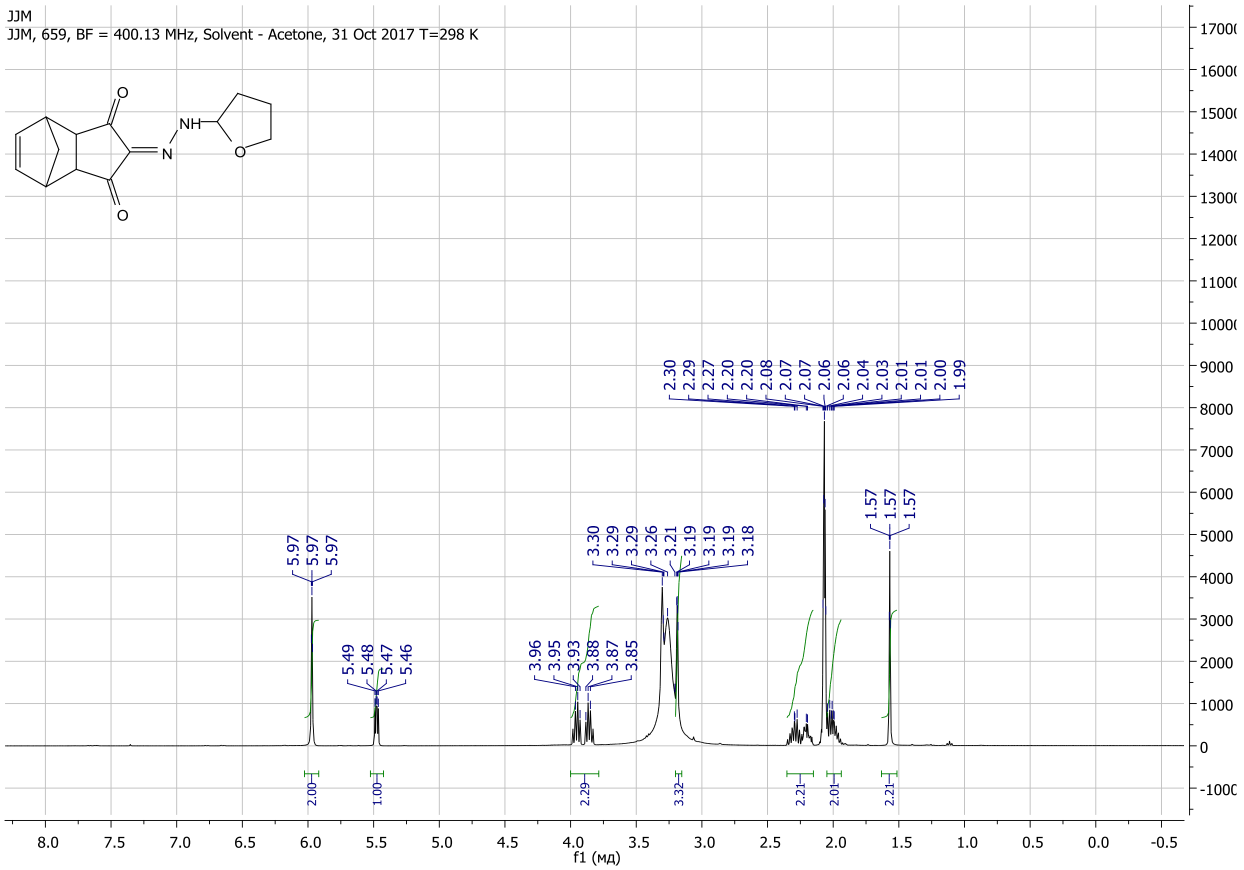

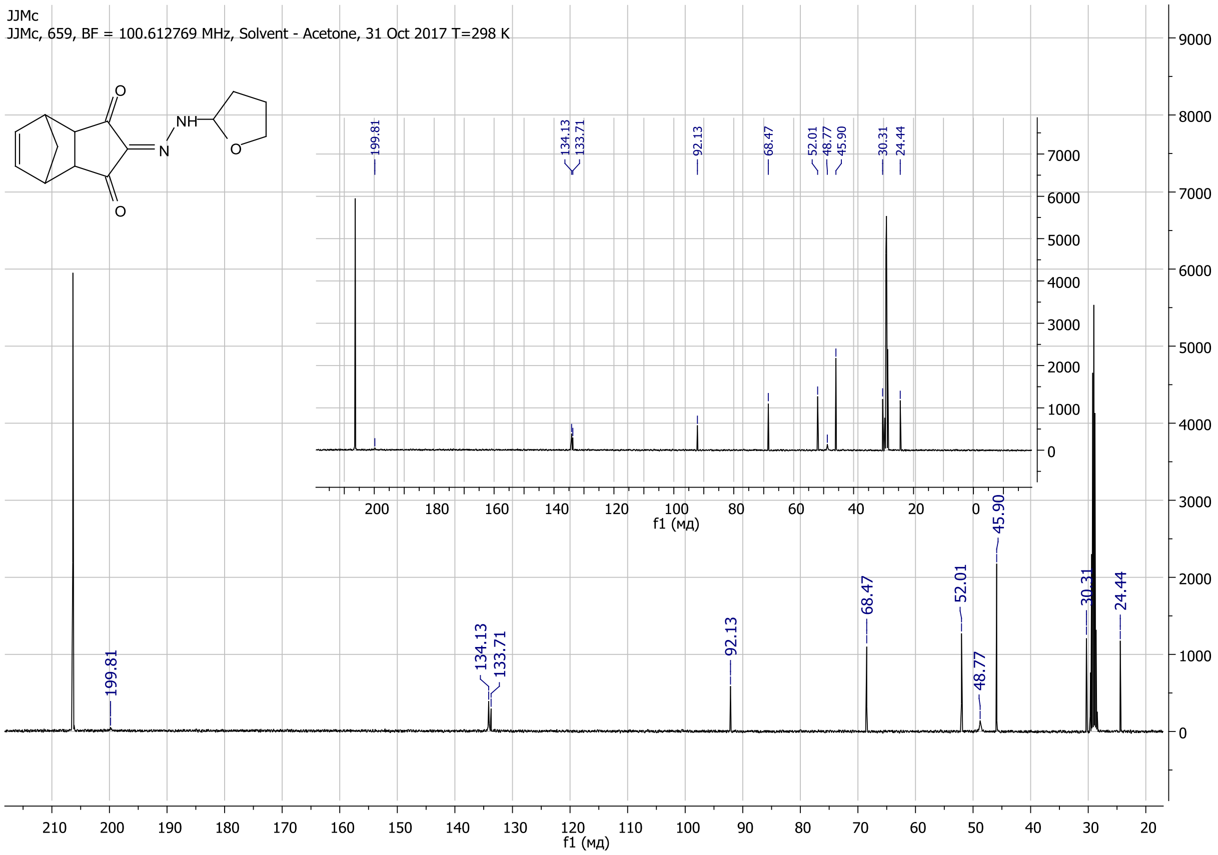

^{1}H and ^{13}C NMR of compound **2c**

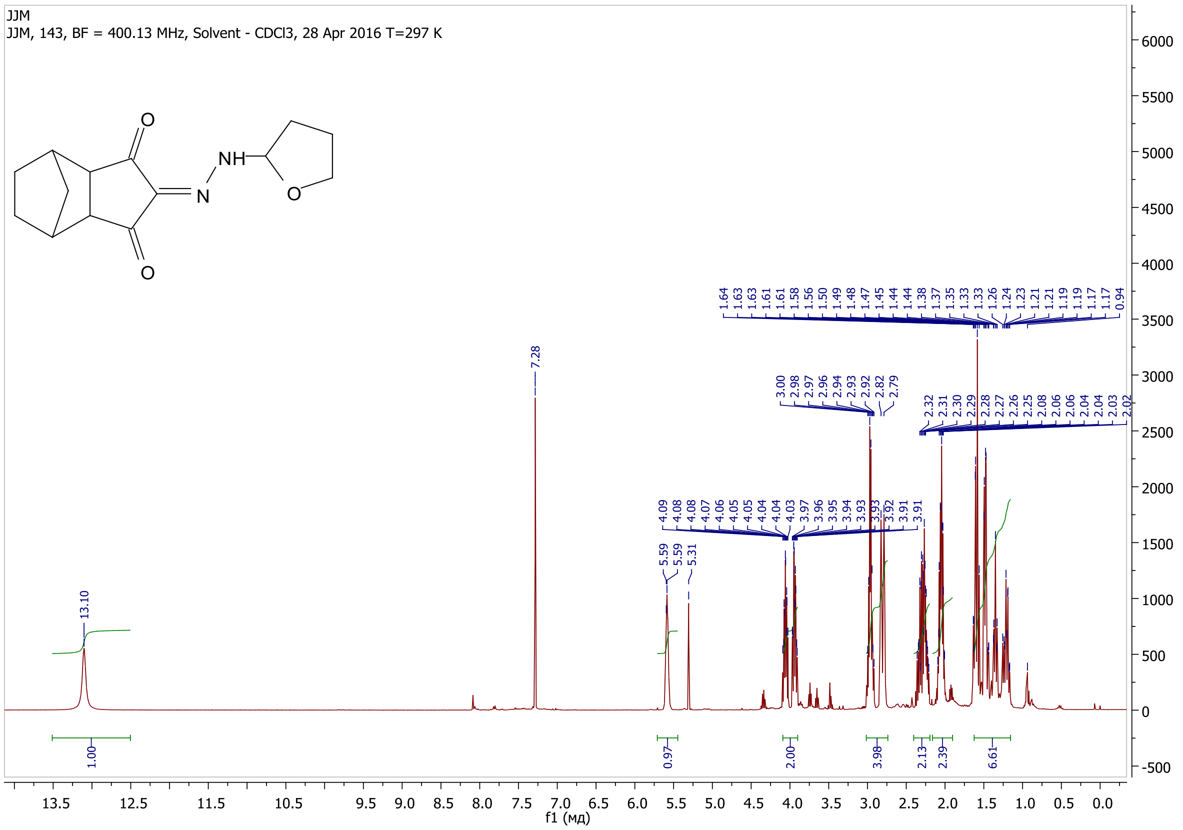

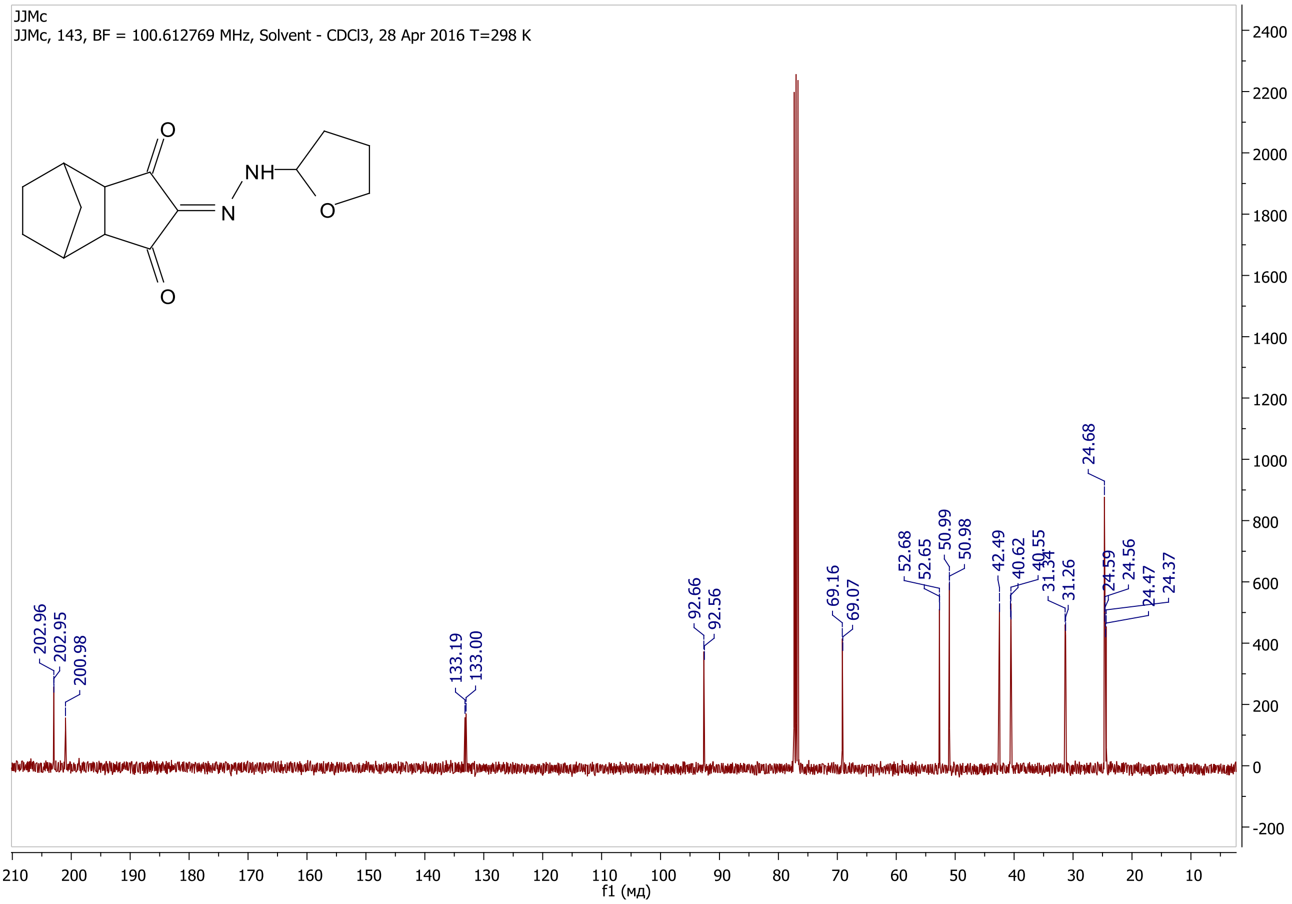

^{1}H and ^{13}C NMR of compound **2d**

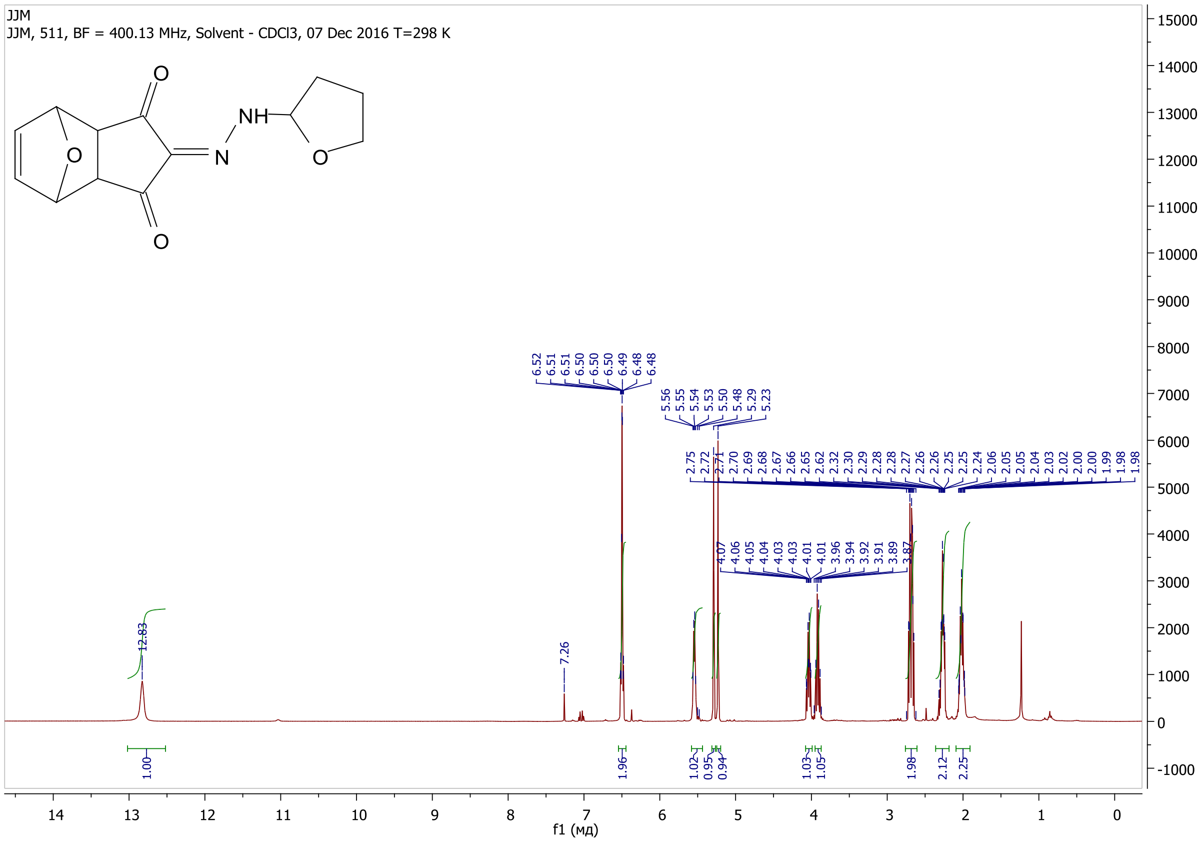

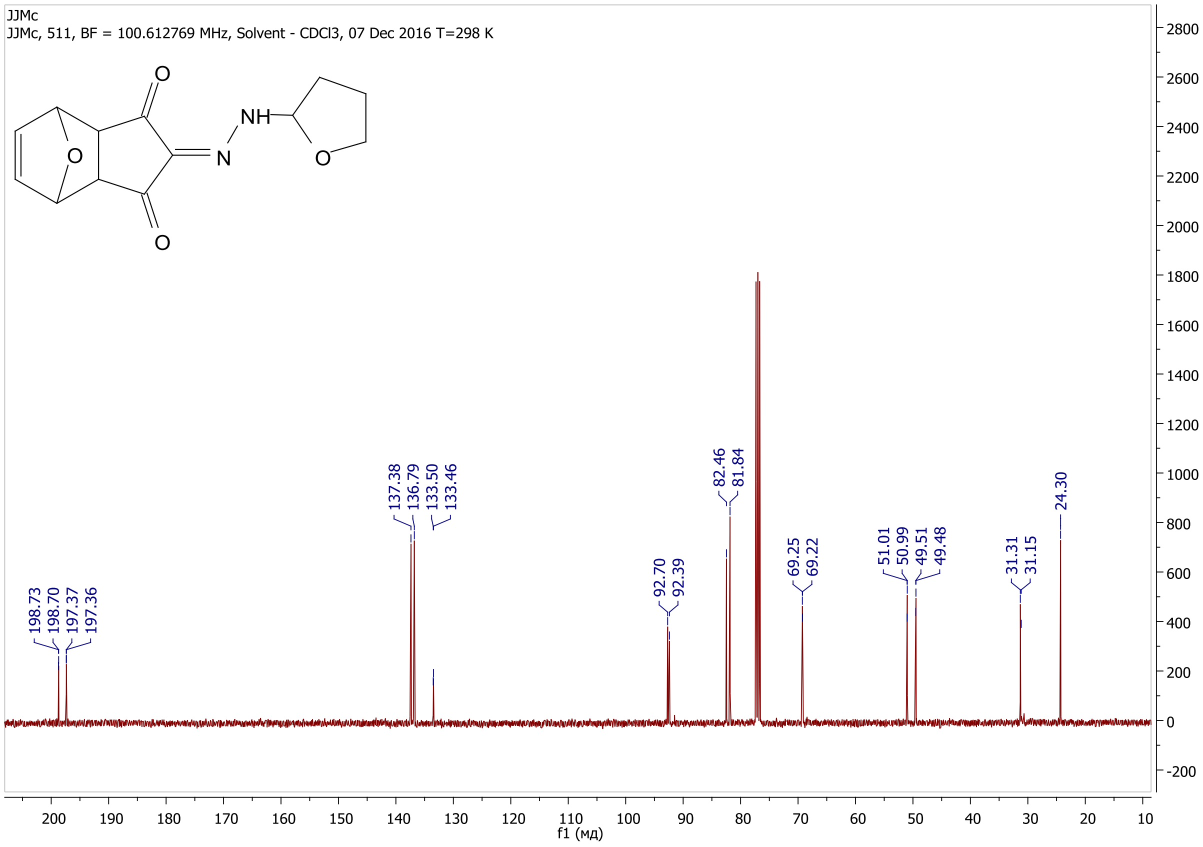

^{1}H and ^{13}C NMR of compound **2f**

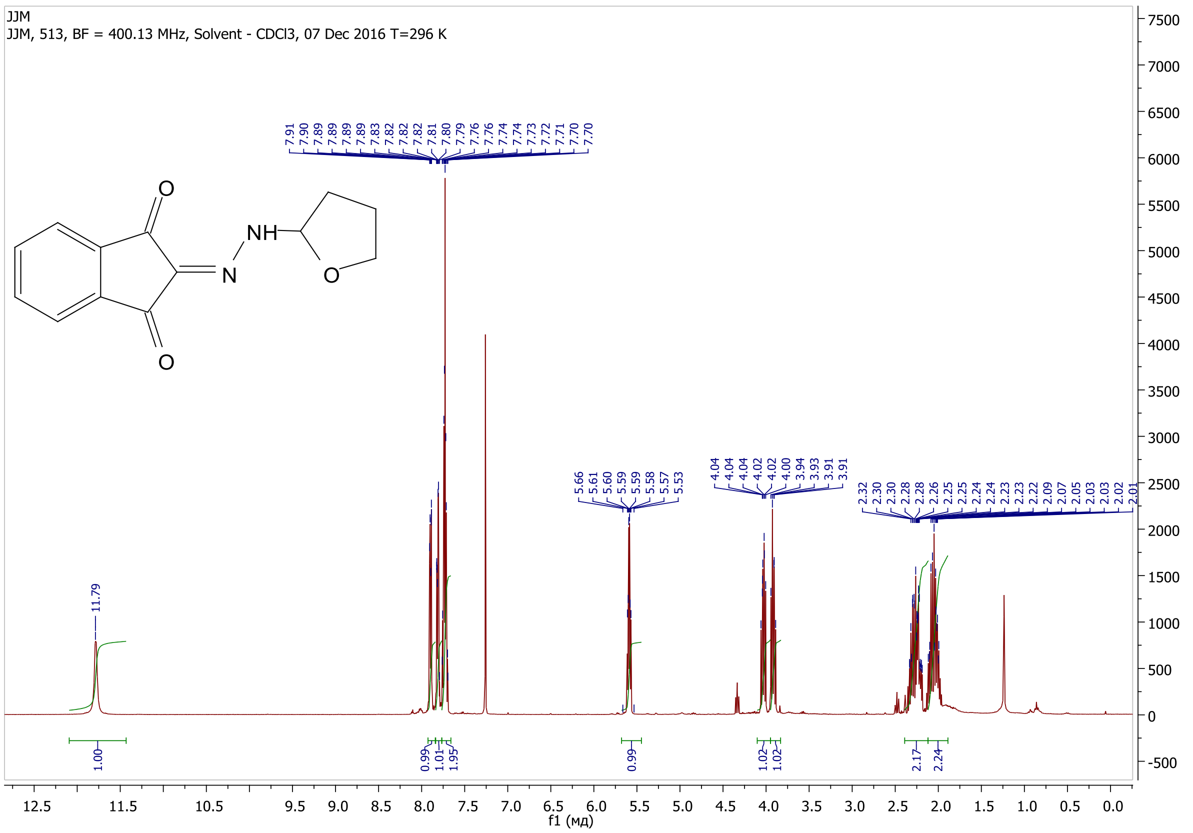

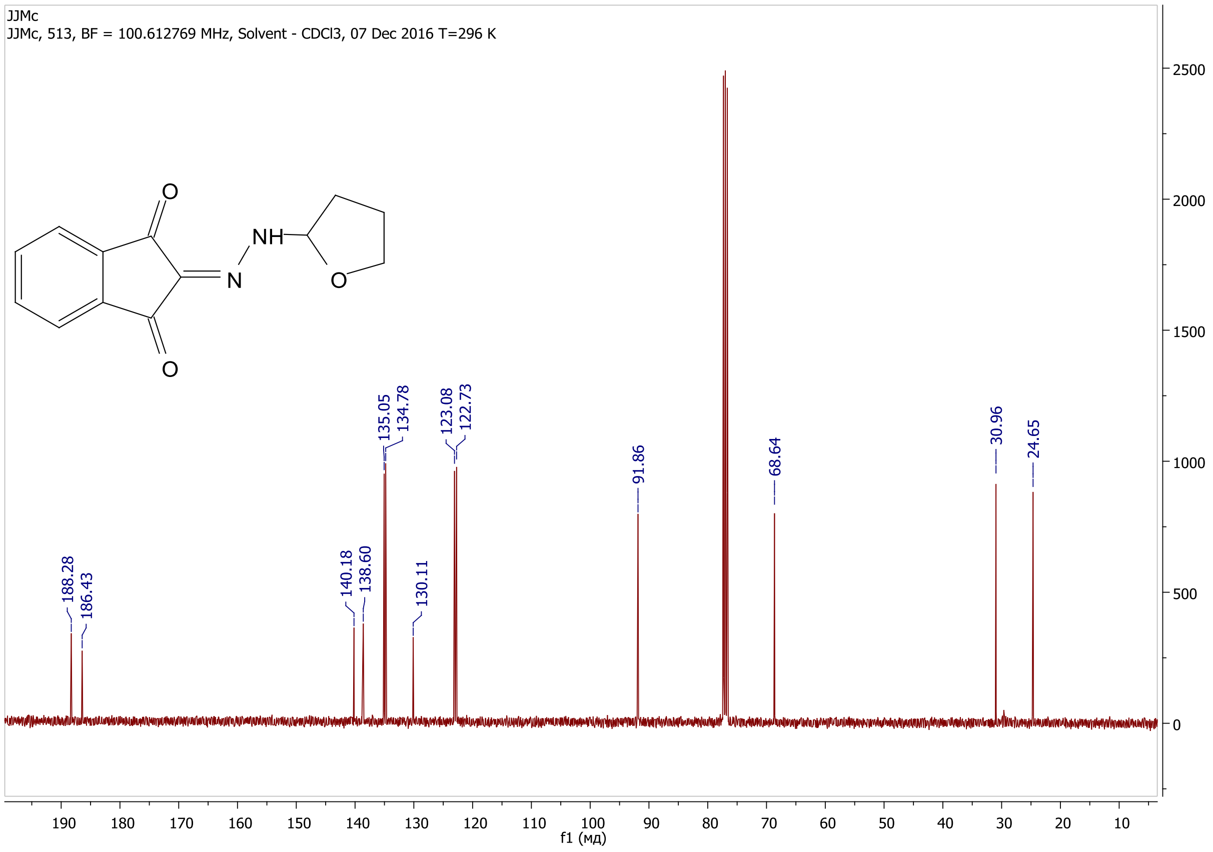

^{1}H and ^{13}C NMR of compound **3**

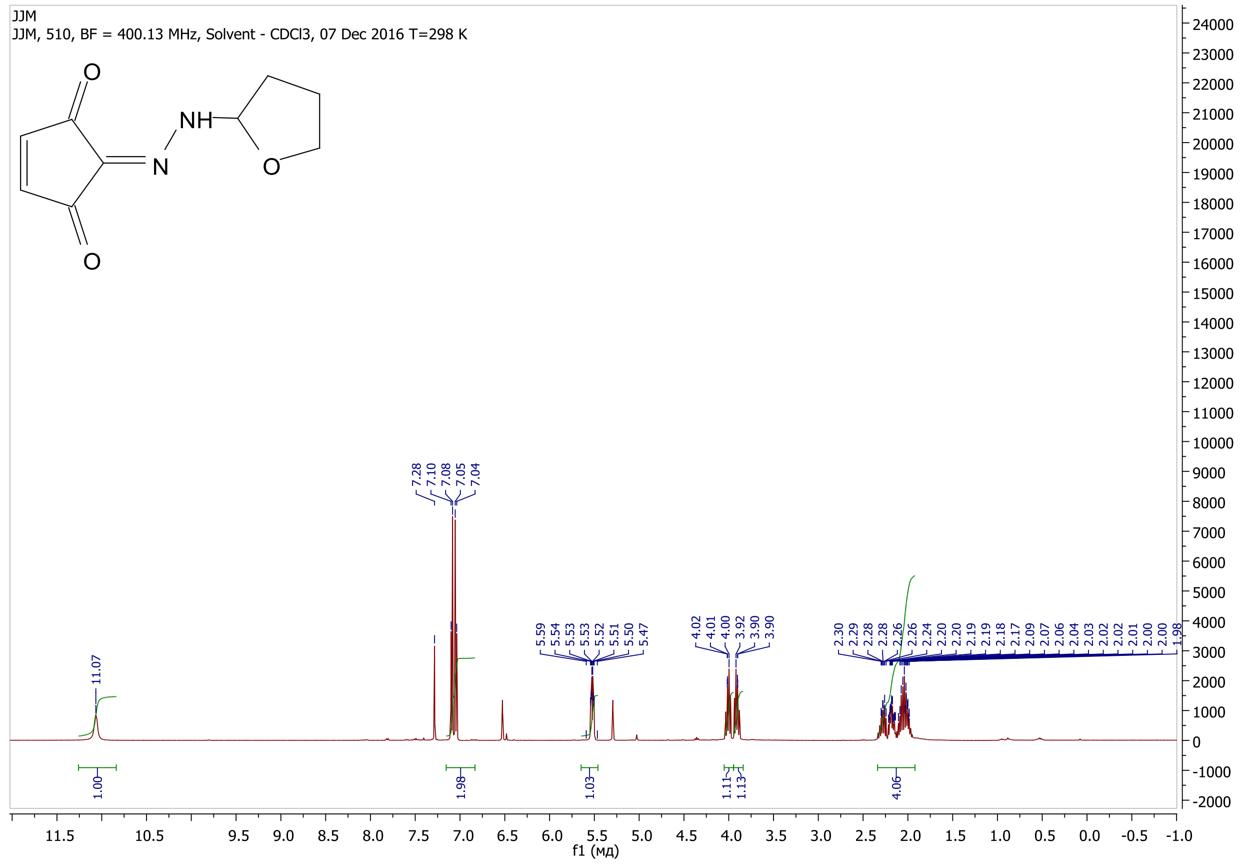

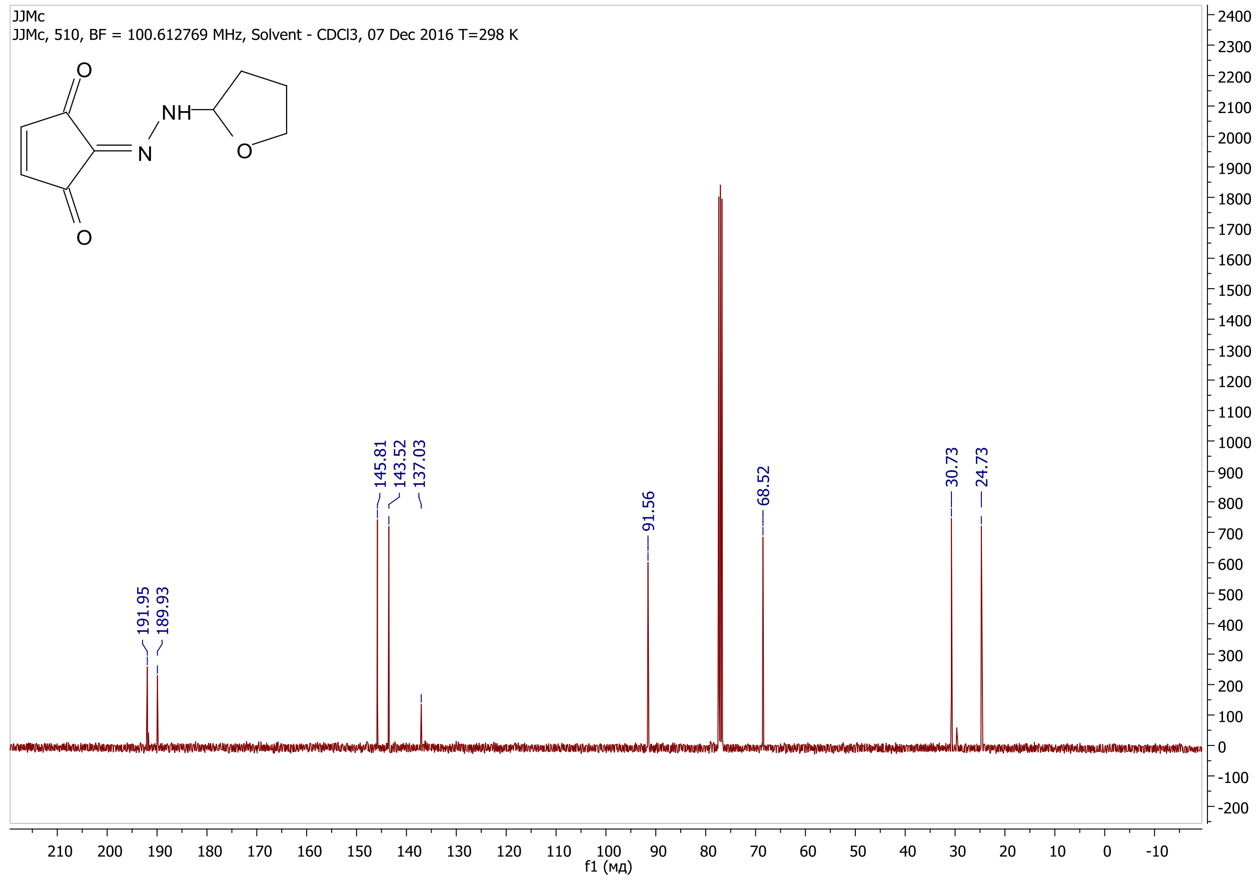

^{1}H and ^{13}C NMR of compound **5b**

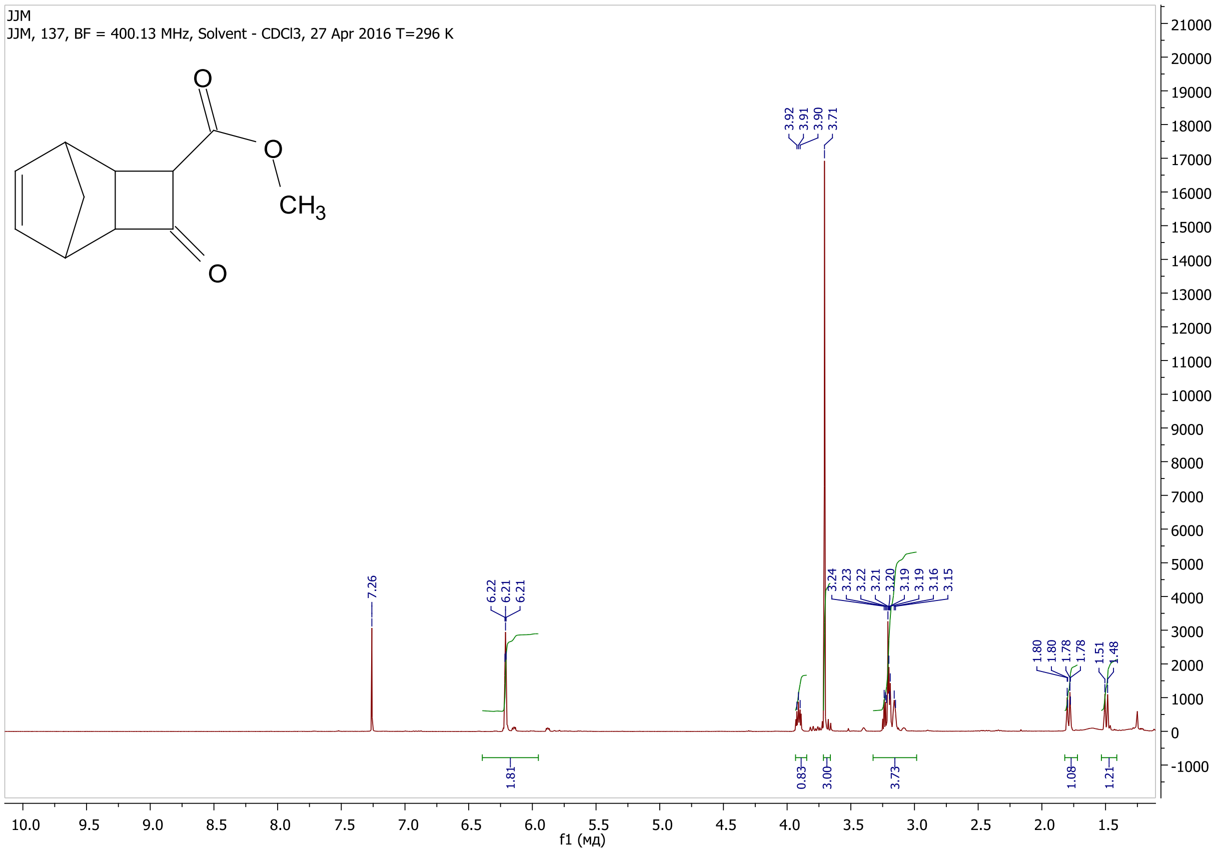

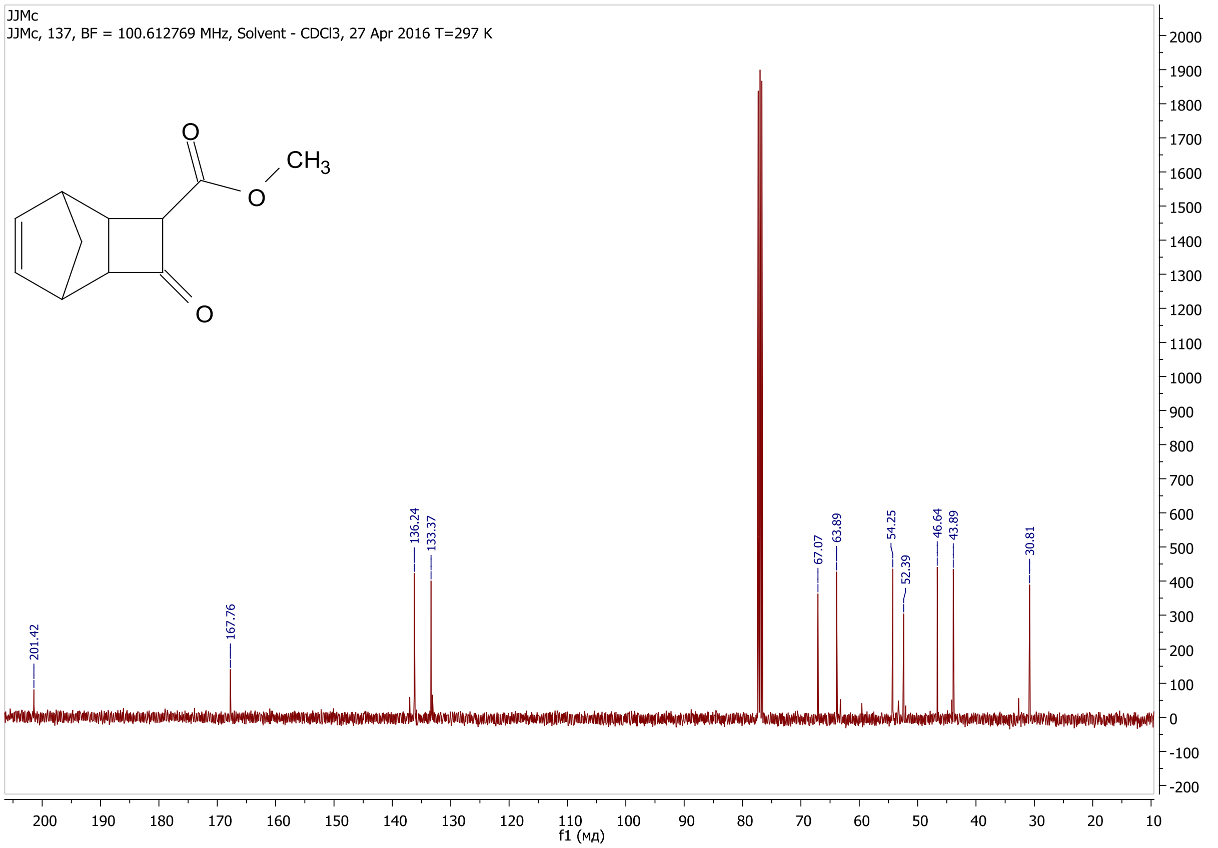

^{1}H and ^{13}C NMR of compound **5c**

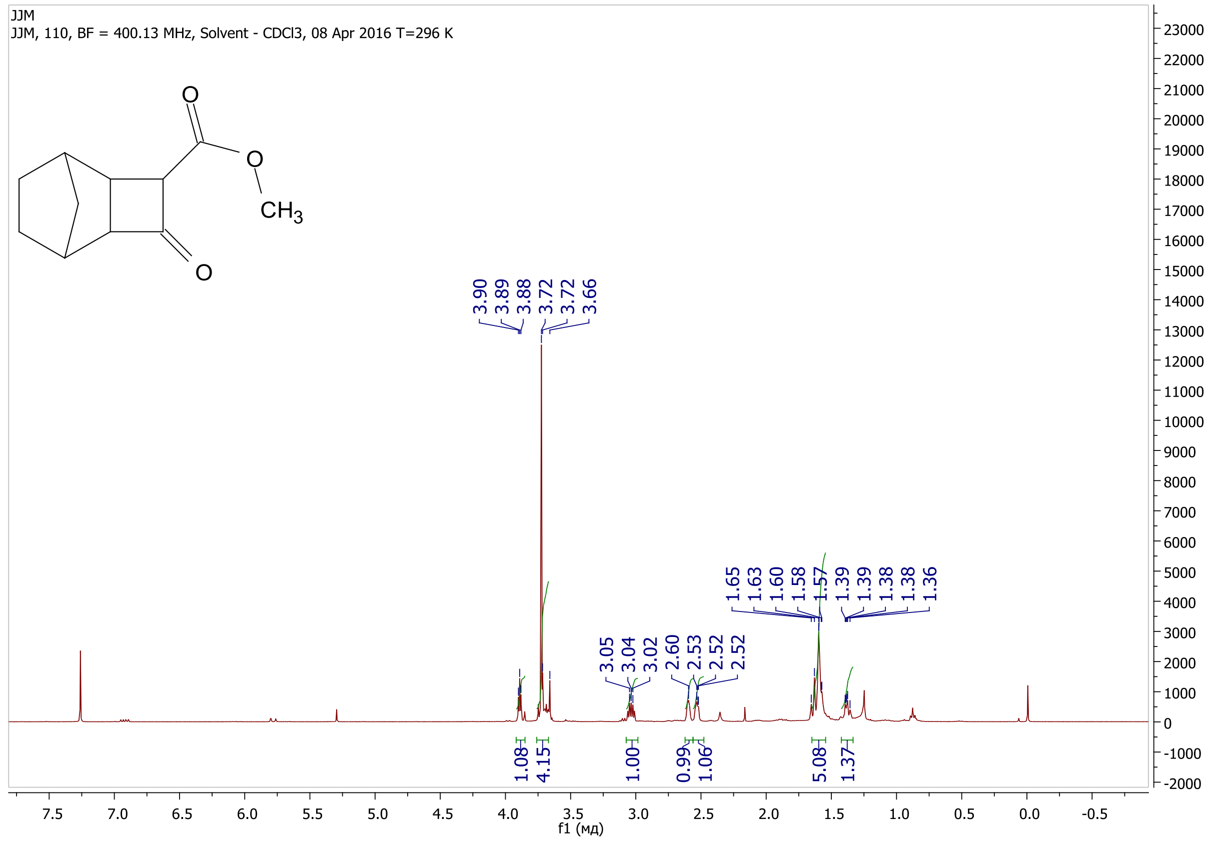

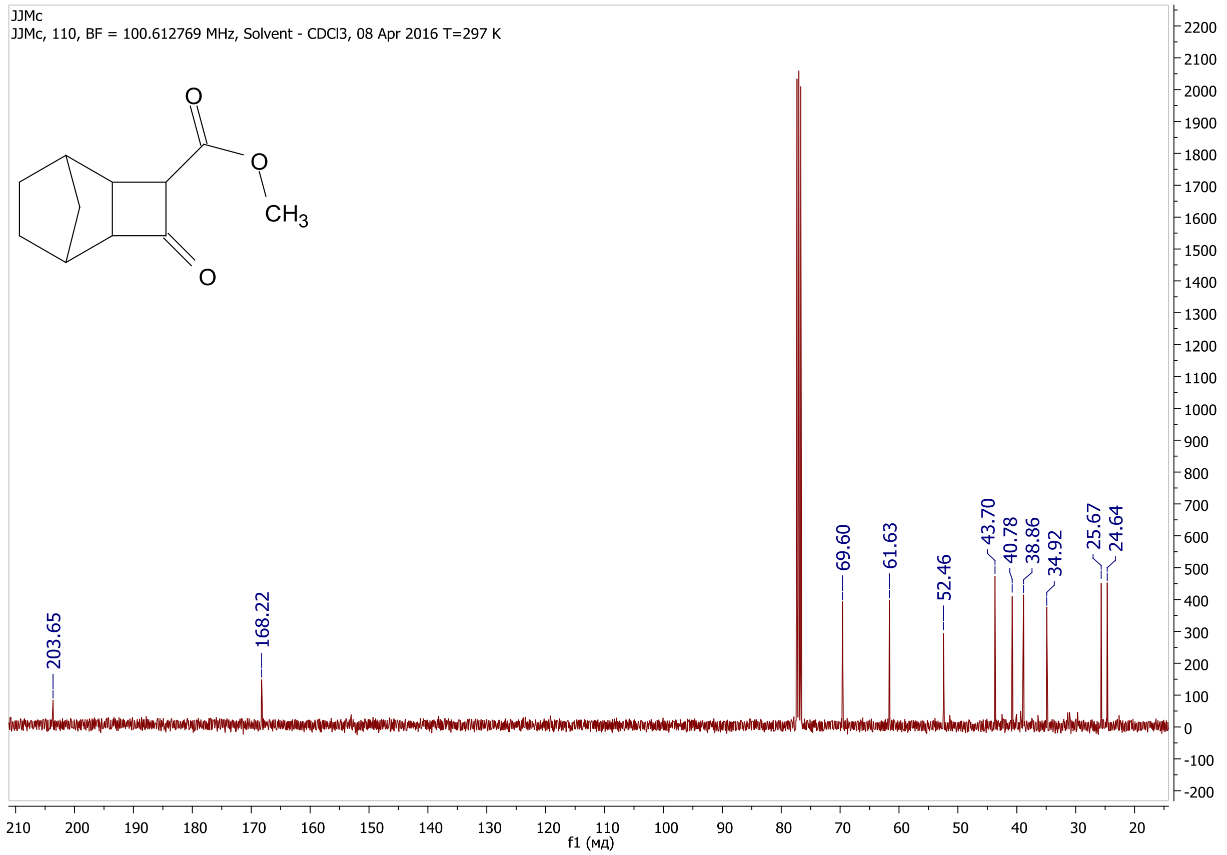

checkCIF/PLATON report (X-Ray data for compound 2b)

You have not supplied any structure factors. As a result the full set of tests cannot be run.

THIS REPORT IS FOR GUIDANCE ONLY. IF USED AS PART OF A REVIEW PROCEDURE FOR PUBLICATION, IT SHOULD NOT REPLACE THE EXPERTISE OF AN EXPERIENCED CRYSTALLOGRAPHIC REFEREE.

No syntax errors found. CIF dictionary Interpreting this report

Datablock: jjm_i1

```
Bond precision:  C-C = 0.0031 A          Wavelength=0.71073

Cell:            a=7.6262(5)     b=17.4605(10)        c=9.4488(6)
                 alpha=90        beta=101.673(6)      gamma=90
Temperature:     100 K

                Calculated                 Reported
Volume          1232.16(14)                1232.15(13)
Space group     P 21/c                     P 1 21/c 1
Hall group      -P 2ybc                    -P 2ybc
Moiety formula  C14 H16 N2 O3              C14 H16 N2 O3
Sum formula     C14 H16 N2 O3              C14 H16 N2 O3
Mr              260.29                     260.29
Dx,g cm-3       1.403                      1.403
Z               4                          4
Mu (mm-1)       0.100                      0.100
F000            552.0                      552.0
F000'           552.26
h,k,lmax        9,22,12                    9,22,12
Nref            2831                       2817
Tmin,Tmax       0.976,0.980                0.854,1.000
Tmin'           0.975

Correction method= # Reported T Limits: Tmin=0.854
Tmax=1.000 AbsCorr = MULTI-SCAN

Data completeness= 0.995          Theta(max)= 27.493

R(reflections)= 0.0582( 2247)     wR2(reflections)= 0.1519( 2817)

S = 1.073                  Npar= 172
```

The following ALERTS were generated. Each ALERT has the format
test-name_ALERT_alert-type_alert-level.
Click on the hyperlinks for more details of the test.

Alert level B

PLAT097_ALERT_2_B Large Reported Max. (Positive) Residual Density 0.86 eA-3

Alert level C

DIFMX02_ALERT_1_C The maximum difference density is > 0.1*ZMAX*0.75 The relevant atom site should be identified.
PLAT094_ALERT_2_C Ratio of Maximum / Minimum Residual Density .... 2.76 Report

Alert level G

PLAT007_ALERT_5_G Number of Unrefined Donor-H Atoms .... 1 Report PLAT398_ALERT_2_G Deviating C-O-C Angle From 120 for O2 107.9 Degree PLAT793_ALERT_4_G Model has Chirality at C9 (Centro SPGR) S Verify PLAT793_ALERT_4_G Model has Chirality at C10 (Centro SPGR) R Verify PLAT793_ALERT_4_G Model has Chirality at C12 (Centro SPGR) S Verify PLAT793_ALERT_4_G Model has Chirality at C14 (Centro SPGR) S Verify PLAT793_ALERT_4_G Model has Chirality at C16 (Centro SPGR) R Verify PLAT933_ALERT_2_G Number of OMIT Records in Embedded .res File ... 3 Note

0 **ALERT level A** = Most likely a serious problem - resolve or explain
1 **ALERT level B** = A potentially serious problem, consider carefully
2 **ALERT level C** = Check. Ensure it is not caused by an omission or oversight
8 **ALERT level G** = General information/check it is not something unexpected

```
1 ALERT type 1 CIF construction/syntax error, inconsistent or missing data
4 ALERT type 2 Indicator that the structure model may be wrong or deficient
0 ALERT type 3 Indicator that the structure quality may be low
5 ALERT type 4 Improvement, methodology, query or suggestion
1 ALERT type 5 Informative message, check
```

It is advisable to attempt to resolve as many as possible of the alerts in all categories. Often the minor alerts point to easily fixed oversights, errors and omissions in your CIF or refinement strategy, so attention to these fine details can be worthwhile. In order to resolve some of the more serious problems it may be necessary to carry out additional measurements or structure refinements. However, the purpose of your study may justify the reported deviations and the more serious of these should normally be commented upon in the discussion or experimental section of a paper or in the "special_details" fields of the CIF. checkCIF was carefully designed to identify outliers and unusual parameters, but every test has its limitations and alerts that are not important in a particular case may appear. Conversely, the absence of alerts does not guarantee there are no aspects of the results needing attention. It is up to the individual to critically assess their own results and, if necessary, seek expert advice.

Publication of your CIF in IUCr journals

A basic structural check has been run on your CIF. These basic checks will be run on all CIFs submitted for publication in IUCr journals (*Acta Crystallographica*, *Journal of Applied Crystallography*, *Journal of Synchrotron Radiation*); however, if you intend to submit to *Acta Crystallographica Section C* or *E* or *IUCrData*, you should make sure that full publication checks are run on the final version of your CIF prior to submission.

Publication of your CIF in other journals

Please refer to the *Notes for Authors* of the relevant journal for any special instructions relating to CIF submission.

PLATON version of 09/11/2017; check.def file version of 08/11/2017

Datablock jjm_i1 - ellipsoid plot

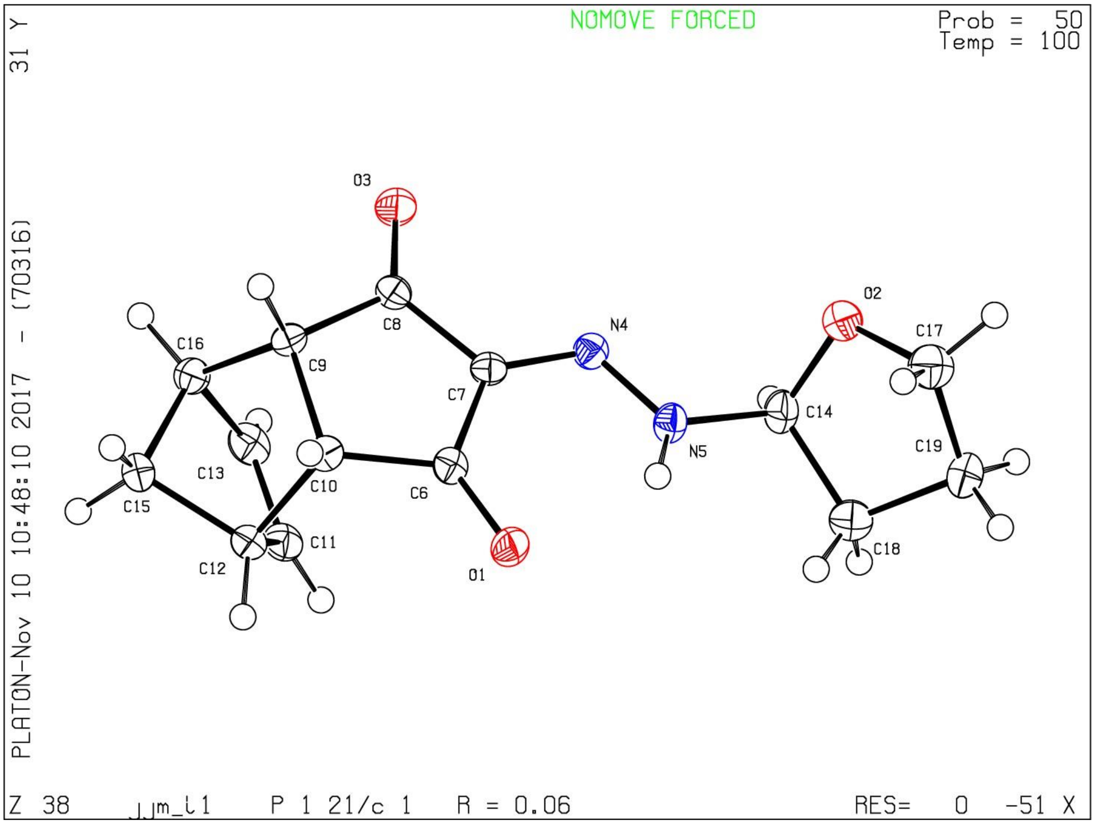

checkCIF/PLATON report (X-Ray data for compound 1c)

You have not supplied any structure factors. As a result the full set of tests cannot be run.

THIS REPORT IS FOR GUIDANCE ONLY. IF USED AS PART OF A REVIEW PROCEDURE FOR PUBLICATION, IT SHOULD NOT REPLACE THE EXPERTISE OF AN EXPERIENCED CRYSTALLOGRAPHIC REFEREE.

No syntax errors found. CIF dictionary Interpreting this report

Datablock: 103-5182-4058_jjm_092

```
Bond precision:  C-C = 0.0016 A          Wavelength=0.71073

Cell:             a=9.6650(4)      b=9.3156(4)      c=19.4448(9)
                  alpha=90         beta=90          gamma=90
Temperature:      100 K

                Calculated                    Reported
Volume          1750.72(13)                   1750.73(14)
Space group     P b c a                       P b c a
Hall group      -P 2ac 2ab                    -P 2ac 2ab
Moiety formula  C10 H10 N2 O2                 C10 H10 N2 O2
Sum formula     C10 H10 N2 O2                 C10 H10 N2 O2
Mr              190.20                        190.20
Dx,g cm-3       1.443                         1.443
Z               8                             8
Mu (mm-1)       0.103                         0.103
F000            800.0                         800.0
F000'           800.37
h,k,lmax        12,12,25                      12,12,25
Nref            2010                          2010
Tmin,Tmax                                     0.755,1.000
Tmin'

Correction method= # Reported T Limits: Tmin=0.755 Tmax=1.000
AbsCorr = MULTI-SCAN

Data completeness= 1.000          Theta(max)= 27.494

R(reflections)= 0.0364( 1755)     wR2(reflections)= 0.0969( 2010)

S = 1.059                 Npar= 127
```

The following ALERTS were generated. Each ALERT has the format
test-name_ALERT_alert-type_alert-level.
Click on the hyperlinks for more details of the test.

Alert level B

PLAT230_ALERT_2_B Hirshfeld Test Diff for N1 --C2 .. 7.1 s.u.

Alert level C

CRYSC01_ALERT_1_C No recognised colour has been given for crystal colour. PLAT053_ALERT_1_C Minimum Crystal Dimension Missing (or Error) ... Please Check
PLAT054_ALERT_1_C Medium Crystal Dimension Missing (or Error) ... Please Check
PLAT055_ALERT_1_C Maximum Crystal Dimension Missing (or Error) ... Please Check

Alert level G

```
PLAT793_ALERT_4_G Model has Chirality at C4          (Centro SPGR)          R Verify
PLAT793_ALERT_4_G Model has Chirality at C5          (Centro SPGR)          R Verify
PLAT793_ALERT_4_G Model has Chirality at C8          (Centro SPGR)          S Verify
PLAT793_ALERT_4_G Model has Chirality at C9          (Centro SPGR)          S Verify
```

0 **ALERT level A** = Most likely a serious problem - resolve or explain
1 **ALERT level B** = A potentially serious problem, consider carefully
4 **ALERT level C** = Check. Ensure it is not caused by an omission or oversight
4 **ALERT level G** = General information/check it is not something unexpected

```
4 ALERT type 1 CIF construction/syntax error, inconsistent or missing data
1 ALERT type 2 Indicator that the structure model may be wrong or deficient
0 ALERT type 3 Indicator that the structure quality may be low
4 ALERT type 4 Improvement, methodology, query or suggestion
0 ALERT type 5 Informative message, check
```

It is advisable to attempt to resolve as many as possible of the alerts in all categories. Often the minor alerts point to easily fixed oversights, errors and omissions in your CIF or refinement strategy, so attention to these fine details can be worthwhile. In order to resolve some of the more serious problems it may be necessary to carry out additional measurements or structure refinements. However, the purpose of your study may justify the reported deviations and the more serious of these should normally be commented upon in the discussion or experimental section of a paper or in the "special_details" fields of the CIF. checkCIF was carefully designed to identify outliers and unusual parameters, but every test has its limitations and alerts that are not important in a particular case may appear. Conversely, the absence of alerts does not guarantee there are no aspects of the results needing attention. It is up to the individual to critically assess their own results and, if necessary, seek expert advice.

Publication of your CIF in IUCr journals

A basic structural check has been run on your CIF. These basic checks will be run on all CIFs submitted for publication in IUCr journals (*Acta Crystallographica*, *Journal of Applied Crystallography*, *Journal of Synchrotron Radiation*); however, if you intend to submit to *Acta Crystallographica Section C* or *E* or *IUCrData*, you should make sure that full publication checks are run on the final version of your CIF prior to submission.

Publication of your CIF in other journals

Please refer to the *Notes for Authors* of the relevant journal for any special instructions relating to CIF submission.

PLATON version of 09/11/2017; check.def file version of 08/11/2017

Datablock 103-5182-4058_jjm_092 - ellipsoid plot

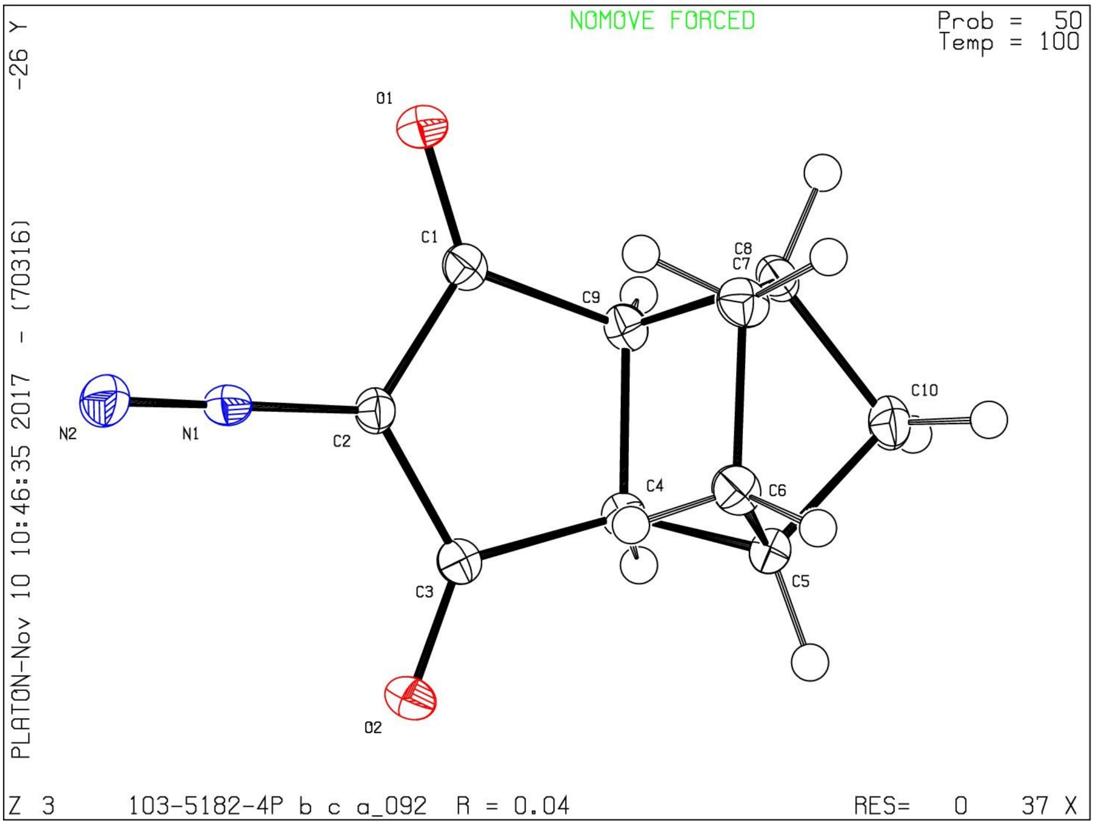

Supplement: File 1 — NMR spectra of all new compounds and data of X-ray analysis for compounds 1c (CCDC 1584937) and 2b (CCDC 1584938). [file Beilstein_J_Org_Chem-14-2250-s001.pdf]
